# RSPO2 Coordinates with GDF9:BMP15 Heterodimers to Promote Granulosa Cell and Oocyte Development in Mice

**DOI:** 10.1002/advs.202501973

**Published:** 2025-06-10

**Authors:** Yingmei Wang, Hongjiang Li, Liji You, Shuhui Wang, Jinglei Bie, Ziyang Su, Lanying Shi, You‐Qiang Su

**Affiliations:** ^1^ Shandong Provincial Key Laboratory of Animal Cell and Developmental Biology School of Life Sciences Shandong University Qingdao 266237 China

**Keywords:** CTNNB1, female fertility, follicles, GDFP:BMP15 heterodimer, granulosa cells, oocyte‐granulosa cell communication, oocytes, OSF, RSPO2, SMAD2

## Abstract

The generation of mature oocytes, a cornerstone of reproduction, relies on the coordinated interactions between oocytes and surrounding follicular somatic cells. Central to this process is the bidirectional communication between the oocyte and granulosa cells, mediated by oocyte‐secreted factors (OSFs), including GDF9 and BMP15. While GDF9 and BMP15 are well‐established regulators of oocyte and follicle development, the role of additional OSFs and their coordination with GDF9 and BMP15 remains largely unclear. Here, RSPO2 is identified as a key OSF that coordinates with the GDF9:BMP15 heterodimer to regulate granulosa cell development and enhance oocyte competence. RSPO2, primarily expressed in oocytes, interacts with GDF9:BMP15 to preserve transcriptomic integrity in preantral granulosa cells. This coordination is gene‐specific, exhibiting either synergistic or antagonistic effects depending on the target genes, and involves crosstalk between CTNNB1‐ and SMAD2‐dependent pathways. Conditional knockout of *Rspo2* in oocytes causes severe defects in granulosa cell and oocyte development, leading to subfertility and earlier reproductive lifespan termination. Transcriptomic analysis shows that RSPO2 loss disrupts key granulosa cell genes (e.g., *Amh, Ccnd2, Inhbb, Kitl*) and compromises oocyte mitochondrial function, reducing developmental competence. These findings establish RSPO2 as an essential factor in the oocyte‐granulosa cell regulatory loop, crucial for ovarian function and fertility.

## Graphical abstract

RSPO2 and GDF9:BMP15 heterodimers—core components of oocyte‐secreted factors (OSFs)—coordinate to shape the molecular architecture of preantral granulosa cells via gene‐specific synergistic and antagonistic regulation, mediated through CTNNB1–SMAD2 signaling crosstalk. Using an oocyte‐specific *Rspo2* knockout mouse model, this study demonstrates that such coordinated action on granulosa cells is essential for granulosa cell development, oocyte competence and female fertility.

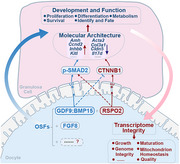

## 1 Introduction

The generation of functionally competent, mature oocytes is fundamental to successful reproduction. However, this process is remarkably complex, requiring a finely orchestrated interplay between systemic signals and local microenvironmental cues.^[^ ^1^ ^]^ The process begins with the selective activation of a subset of dormant primordial follicles from a perinatally established storage pool. This pool consists of a finite number of non‐growing oocytes arrested at the diplotene stage of the first meiotic prophase and enclosed by a single layer of flattened pregranulosa cells. These primordial follicles constitute the ovarian reserve, and their gradual depletion leads to follicular exhaustion and reproductive senescence. Upon activation, the oocyte increases in size, while the surrounding granulosa cells transition to a cuboidal shape and proliferate mitotically. Together, they undergo a highly coordinated developmental program, progressing through the primary and secondary follicle stages before reaching the antral follicle stage.

In antral follicles, the formation of the antrum—a fluid‐filled cavity—divides the granulosa cells into two structurally and functionally distinct populations: mural granulosa cells, which line the inner follicle wall and primarily perform endocrine functions, and cumulus cells, which surround the oocyte and provide essential metabolic and molecular support.^[^ ^1c^ ^]^ These structural and functional differences arise from transcriptomic divergence.^[^ ^2^ ^]^ While preantral follicle development primarily depends on locally produced growth factors, antral follicle maturation becomes strictly dependent on pituitary‐derived follicle‐stimulating hormone (FSH).^[^ ^1d^ ^]^ Eventually, under the influence of the mid‐estrous cycle surge of luteinizing hormone (LH), oocytes resume and complete the first meiotic division, becoming mature oocytes. They are then released from the follicles and expelled into the oviduct, aided by the expanded cumulus cells, while mural granulosa cells undergo terminal differentiation and contribute to corpus luteum formation.^[^ ^3^ ^]^ Throughout follicular development, oocytes maintain direct physical contact with granulosa cells via specialized filopodia known as transzonal projections (TZPs), which extend from granulosa cells to the oocyte surface and form heterologous gap junctions between the two cell types.^[^ ^4^ ^]^ Simultaneously, dynamic oocyte microvilli bud from the oolemma, creating specialized structures that facilitate the release of oocyte‐secreted factors (OSFs) to regulate granulosa cell activity.^[^ ^5c^ ^]^ Together, TZPs and microvilli establish the structural basis for oocyte–granulosa cell interactions, facilitating the exchange of metabolic substrates and regulatory signals essential for coordinated follicular and oocyte development.^[^ ^5^ ^]^

At the core of this intricate process of mature oocyte generation is the bidirectional communication between the oocyte and its surrounding granulosa cells.^[^ ^1^, ^5^ ^]^ This communication begins during primordial follicle assembly, persists throughout follicular development, and concludes upon ovulation. Granulosa cells support oocyte development by promoting growth, maintaining meiotic arrest, triggering meiotic resumption, and enhancing developmental competence. In turn, the oocyte actively regulates granulosa cell proliferation, differentiation, steroidogenesis, metabolism, and survival. This dynamic interplay ensures the synchronized development of both cell types, which is essential for proper follicle maturation and oocyte competence. Disruptions in this communication network can impair folliculogenesis, contributing to reproductive disorders such as premature ovarian insufficiency (POI), polycystic ovary syndrome (PCOS), oocyte maturation defects (OMDs), and ovulation failure.^[^ ^6^ ^]^ Despite its critical role in female fertility, the molecular mechanisms governing oocyte‐granulosa cell crosstalk remain incompletely understood.

Accumulating evidence suggests that the oocyte plays a central role in its interactions with granulosa cells, establishing an “oocyte‐granulosa cell regulatory loop” within the follicle. Through this loop, the oocyte governs granulosa cell development and function, indirectly regulating its own growth and developmental fate.^[^ ^1^, ^5^ ^]^ OSFs mediate most of these regulatory functions. Among them, growth differentiation factor 9 (GDF9) and bone morphogenetic protein 15 (BMP15), both members of the transforming growth factor beta (TGFB) superfamily, are well‐established key OSFs.^[^ ^1^, ^5^ ^]^ Genetic studies and in vitro experiments across mammalian species have demonstrated that GDF9 and BMP15 are indispensable for folliculogenesis. In mice, these factors are exclusively expressed in oocytes, and their genetic deletion disrupts follicular development, compromises oocyte quality, and reduces female fertility.^[^ ^7^ ^]^ Furthermore, GDF9 and BMP15 either act synergistically or form potent heterodimers to promote granulosa cell proliferation and cholesterol biosynthesis,^[^ ^8^ ^]^ while BMP15, in collaboration with fibroblast growth factor 8 (FGF8), another OSF, enhances cumulus cell glycolysis.^[^ ^9^ ^]^ These findings highlight the importance of coordination of OSF components in regulating granulosa cell function and folliculogenesis. However, the full composition of OSFs and the mechanisms through which its components coordinate to regulate follicular development remain largely unexplored.

R‐spondin 2 (RSPO2), a member of the highly conserved RSPO family of secreted proteins, has emerged as a potential key component of OSFs.^[^ ^10^ ^]^ Beyond its well‐established roles in embryonic development, tissue morphogenesis, and tumorigenesis via potentiation of canonical WNT/CTNNB1 (WNT/β‐catenin) signaling, RSPO2 has been implicated in ovarian folliculogenesis.^[^ ^11^ ^]^ In mice, RSPO2 is robustly expressed in oocytes from the primary follicle stage onward.^[^ ^12^ ^]^ Treatment with recombinant RSPO2 in vitro or RSPO2 agonists in vivo promotes the transition of follicles from the primary to the secondary stage.^[^ ^12a^ ^]^ Although homozygous *Rspo2*‐knockout (KO) or footless (ftl) hypomorphic mice die shortly after birth, heterozygous *Rspo2* ^ftl/+^ females exhibit progressive infertility after four months of age, suggesting a role for RSPO2 in female fertility.^[^ ^12^, ^13^ ^]^ Supporting this, prenatal *Rspo2* ^ftl/ftl^ ovaries transplanted under the kidney capsule of wild‐type (WT) athymic female mice exhibit severely impaired folliculogenesis, with most follicles arrested at the primary stage due to defective granulosa cell proliferation, even though oocyte growth continues.^[^ ^14^ ^]^ These findings suggest that oocyte‐secreted RSPO2 is crucial for granulosa cell development, follicular maturation, and the synchronization of oocyte and follicle growth. However, the physiological role of RSPO2 and its potential coordination with other key OSFs, such as GDF9 and BMP15, remain to be fully elucidated.

Here, we identify RSPO2 as a key component of OSFs that regulates ovarian follicle development by coordinating with GDF9 and BMP15. Conditional knockout (cKO) of *Rspo2* specifically in oocytes disrupts this coordination, leading to severe defects in granulosa cell and oocyte development, subfertility, and earlier reproductive lifespan termination. These findings provide new insights into the “oocentric” regulatory mechanisms underlying folliculogenesis, highlighting the essential role of RSPO2 in ensuring the generation of a functionally competent, mature oocyte.

## 2 Results

### 2.1 OSFs are Essential for Maintaining the Transcriptomic Integrity of Preantral Granulosa Cells

**Figure 1:**
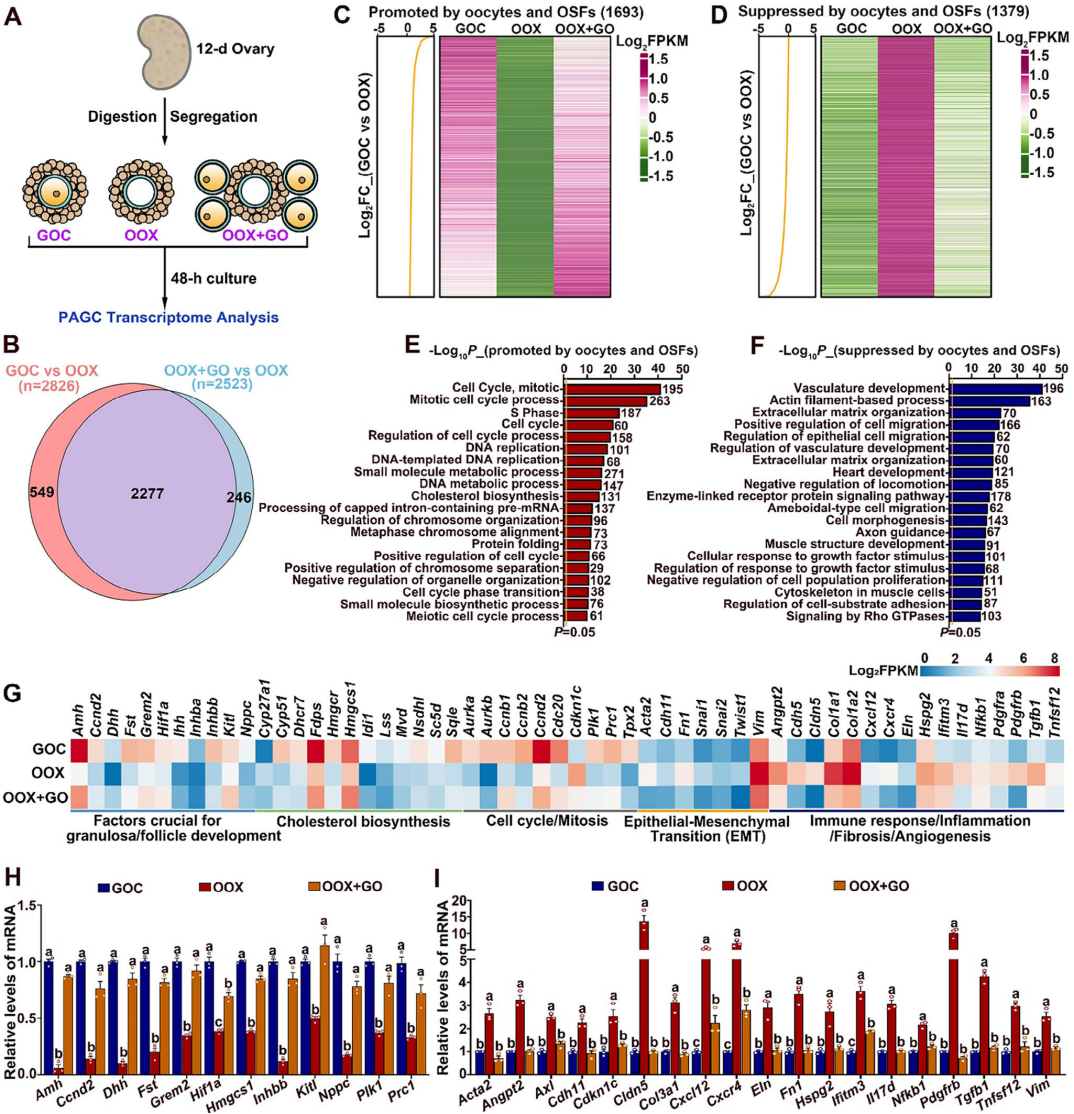
Maintenance of transcriptomic integrity in PAGCs by OSFs. A) Schematic representation of the experimental design used to assess the effect of oocytectomy (OOX) and co‐culture with growing oocytes (GOs) on the transcriptome of PAGCs isolated from 12‐day‐old mouse GOCs. A co‐culture system was established using four GOs and one oocytectomized complex per microliter of culture medium, all isolated from 12‐day‐old mice. B) The Venn diagram showing the overlap DEGs in PAGCs following oocyte removal and co‐culture, respectively. C, D) Heatmaps showing changes in PAGC gene expression in response to oocytes and OSFs, with upregulated genes in (C) and downregulated genes in (D). E, F) Bar graphs showing enriched pathways and biological processes associated with genes upregulated (E) and downregulated (D) by oocytes and OSFs. G) Heatmaps showing expression changes of representative genes regulated by oocytes and OSFs, including those essential for granulosa cell and follicle development, cholesterol biosynthesis, and cell cycle and mitosis, as well as genes invovlved in EMT, immune response, inflammation, fibrosis, and angiogenesis. H, I) qRT‐PCR validation of representative genes from DEGs that are upregulated (H) and downregulated (I) by oocytes and OSFs in OOX‐PAGCs. Experiments were repeated independently three times. Data are presented as Mean ± SEM. Bars marked with different letters indicate significant differenences (*P* < 0.05).

To investigate the effect of oocytes on granulosa cell gene expression in preantral follicles, we performed oocytectomy (OOX) on granulosa cell‐oocyte complexes (GOCs) isolated from secondary follicles of 12‐day‐old female mice. The resulting preantral granulosa cells (PAGCs) were referred to as OOX‐PAGCs. We then assessed transcriptomic changes in these OOX‐PAGCs after 48 h of culture (**Figure** **1**A; Figure S1, Supporting Information). Additionally, we evaluated the impact of the full complement of oocyte‐secreted factors (OSFs), referred to here as the OSF cocktail, on granulosa cell gene expression by co‐culturing the OOX‐PAGCs with growing oocytes isolated from the same‐stage GOCs and analyzing their transcriptome after 48 h (Figure 1A; Figure S1, Supporting Information).

The results showed that OOX and co‐culture with growing oocytes induced differential expression of 2826 and 2523 genes, respectively, in PAGCs (*P* < 0.05, |FC| ≥ 1.5), collectively comprising 3072 unique genes, which we designated as oocyte‐ and OSF cocktail‐regulated genes (Figure 1B; Table S2, Supporting Information). Venn analysis identified 2277 overlapping genes, representing 80.6% of the oocyte‐regulated gene set (Figure 1B). Of the 3072 unique genes, 1693 were promoted, and 1379 were suppressed by oocytes and OSF cocktail, respectively (Figure 1C,D; Table S2, Supporting Information). Gene enrichment analysis revealed that genes promoted by oocytes and OSF cocktails were primarily associated with cell proliferation and metabolism, particularly the “cholesterol biosynthesis” pathway (Figure 1E). Conversely, genes suppressed by oocytes and OSF cocktail were involved in processes such as “vasculature development,” “actin filament‐based processes and cell migration,” “extracellular matrix organization,” and “negative regulation of cell proliferation” (Figure 1F).

Scrutiny of the oocyte‐ and OSF‐regulated gene list revealed a profound downregulation of genes encoding critical regulators of granulosa cell and follicle development—including *Amh*, *Ccnd2*, *Dhh*/*Ihh*, *Fst*, *Grem2*, *Hif1a*, *Inhba*/*Inhbb*, *Kitl*, and *Nppc*—in OOX‐PAGCs (Figure 1G; Table S2, Supporting Information). Conversely, genes implicated in epithelial‐to‐mesenchymal transition (EMT), such as *Acta2* (α‐SMA), *Cdh11* (Cadherin 11/OB‐Cadherin), *Fn1* (fibronectin), *Snai1*, *Snai2* (Slug), *Twist1*, and *Vim* (vimentin), exhibited significant upregulation (Figure 1G; Table S2, Supporting Information). In parallel with EMT‐related changes, OOX‐PAGCs exhibited increased expression of genes linked to immune responses, inflammation, fibrosis, and angiogenesis, including *Angpt2*, *Cdh5*, *Col1a1*, *Col1a2*, *Cxcl12*, *Cxcr4*, *Eln*, *Hspg2*, *Il17d*, *Ifitm3*, *Nfkb1*, *Pdgfra*, *Pdgfrb*, *Tgfb1*, and *Tnfsf12* (Figure 1G; Table S2, Supporting Information). Notably, *Cldn5* — encoding a tight junction component typically associated with Sertoli cells rather than early granulosa cells — was also upregulated (Figure 1G; Table S2, Supporting Information). Strikingly, while genes critical for cell cycle progression/mitosis (e.g., *Aurka*, *Aurkb*, *Ccnb1*, *Ccnb2*, *Ccnd2*, *Cdc20*, *Plk1*, *Prc1*, *Tpx2*) and cholesterol biosynthesis (*Cyp51*, *Dhcr7*, *Fdps*, *Hmgcr*, *Hmgcs1*, *Idi1*, *Lss*, *Mvd*, *Nsdhl*, *Sc5d*, *Sqle*), were downregulated, *Cdkn1c* (an inhibitor of the CCND2‐CDK4 complex) and *Cyp27a1* (a cholesterol‐metabolizing enzyme that converts excess cholesterol to bile acids) were markedly upregulated in OOX‐PAGCs (Figure 1G; Table S2, Supporting Information). Importantly, co‐culture with growing oocytes effectively prevented these transcriptional alterations (Figure 1G; Table S3, Supporting Information). Validation by quantitative real‐time PCR (qRT‐PCR) confirmed the differential expression of representative genes in OOX‐PAGCs and their rescue upon oocyte co‐culture (Figure 1H,I).

### 2.2 RSPO2 Is a Key Component of the OSF Cocktail that Coordinates with GDF9:BMP15 Heterodimers to Regulate Gene Expression in PAGCs

To identify additional potential OSFs involved in the oocyte‐granulosa cell dialogue, we systematically analyzed the annotated functions of proteins encoded by transcripts expressed in oocytes and cumulus cells. This approach led to the identification of several putative ligand‐receptor pairs, with mRNA expression highly enriched in oocytes and cumulus cells, respectively. Among these, *Rspo2* emerged as one of the most abundant oocyte‐expressed ligands, with its cognate receptor, LGR4, preferentially expressed in cumulus cells (Figure S2A,B, Supporting Information). Further analysis of publicly available transcriptomic datasets revealed that *RSPO2* is also robustly expressed in human oocytes (Figure S2B, Supporting Information).

To validate the predominant expression of *Rspo2* in oocytes, we performed qRT‐PCR analyses. The results showed that *Rspo2* was strongly expressed in oocytes compared to other major mouse tissues (Figure S2C, Supporting Information). Within antral follicles, *Rspo2* mRNA levels in oocytes were ≈200 times higher than in mural and cumulus granulosa cells (Figure S2D, Supporting Information). Expression of *Rspo2* mRNA increased dramatically as oocytes grew in primary follicles, reaching its peak in secondary and Graafian follicles (Figure S2E, Supporting Information). The levels of *Rspo2* mRNA remained stable in MII oocytes and pronuclear stage embryos but decreased sharply in embryos at the 2‐cell stage and beyond (Figure S2F, Supporting Information).

Further qRT‐PCR analysis revealed that *Lgr4* was the most abundantly expressed LGR isoform in follicles, with mRNA predominantly found in PAGCs and cumulus cells, and only marginal levels detected in growing or fully‐grown oocytes (FGOs) (Figure S2G, Supporting Information). In contrast, *Lgr5* mRNA was nearly undetectable in both granulosa cells and oocytes, while *Lgr6* was detected exclusively in oocytes, but at very low levels (Figure S2G, Supporting Information). These data collectively indicate that the RSPO2 ligand and its cognate receptor, LGR4, are predominantly expressed in oocytes and their companion granulosa cells, respectively, suggesting the formation of a ligand‐receptor pair that likely functions in follicles, begining at the primary follicle stage.

**Figure 2:**
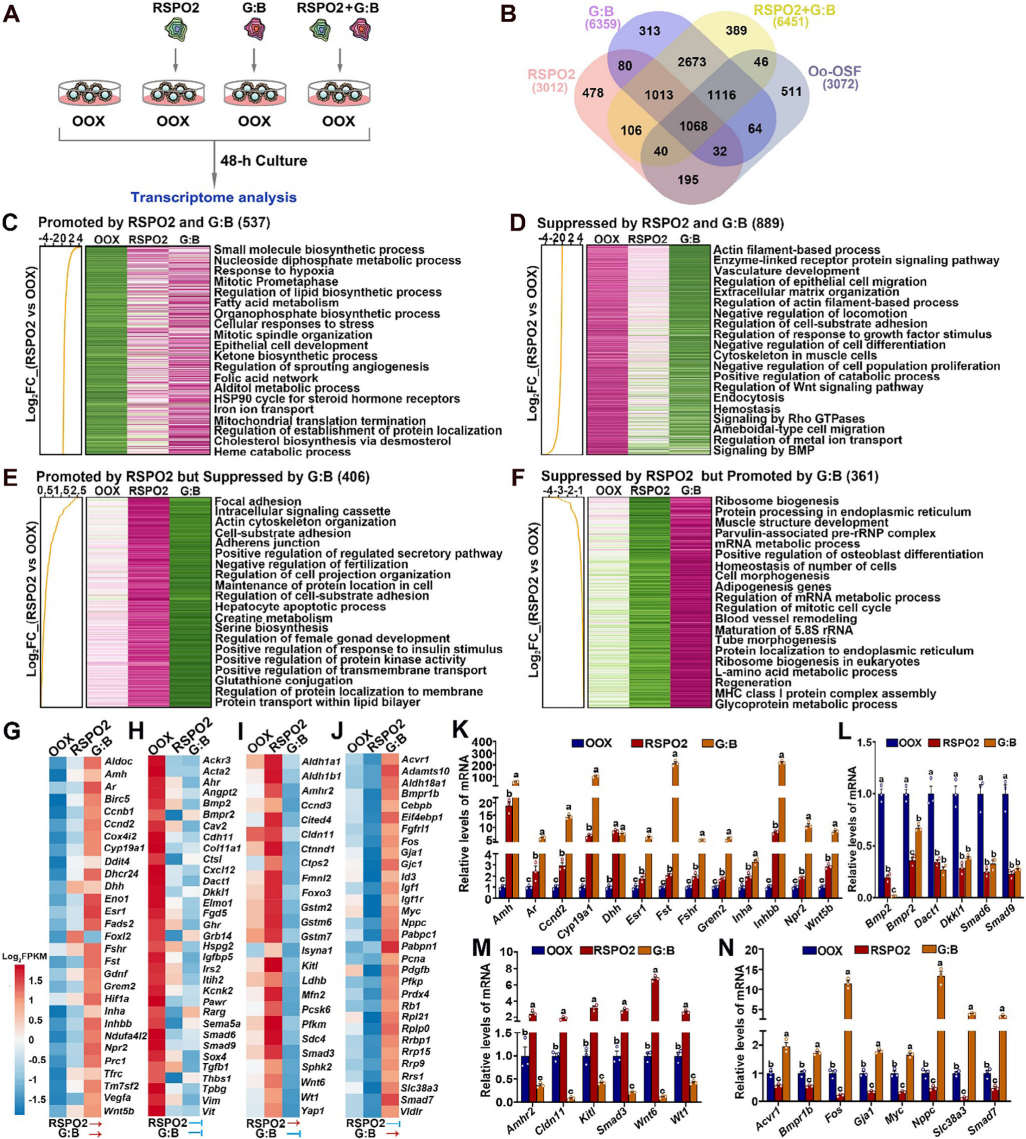
RSPO2 coordinates with GDF9:BMP15 heterodimers to regulate gene expression in PAGCs. A) Schematic diagram illustrating the experimental design to evaluate the effect of RSPO2, GDF9:BMP15 heterodimers (G:B), and RSPO2 + G:B treatments on gene expression in OOX‐PAGCs cultured in vitro. B) Venn diagram showing the overlap of DEGs in OOX‐PAGCs following treatments with RSPO2, G:B, RSPO2 + G:B, or oocyte co‐culture (Oo‐OSF). C–F) Heatmaps showing changes in PAGC gene expression in response to RSPO2 and G:B treatments: (C) genes upregulated by both RSPO2 and G:B; (D) genes downregulated by both RSPO2 and G:B; (E) genes upregulated by RSPO2 but downregulated by G:B; and (F) genes downregulated by RSPO2 but upregulated by G:B. G–J) Heatmaps illustrating expression changes of selected genes in OOX‐PAGCs regulated by RSPO2 and G:B, either synergistically (G, H) or antagonistically (I, J). Arrows (→) denote promoting effects, and bars (⊣) denote suppressive effects. K–N) qRT‐PCR validation of representative genes among DEGs regulated by RSPO2 and G:B either synergistically (K, L) or antagonistically (M,N). Experiments were independently repeated three times. Data are presented as Mean ± SEM. Bars marked with different letters indicate significant differenences (*P* < 0.05).

To investigate whether RSPO2 could be a key component of the OSF cocktail, we performed experiments to determine if RSPO2 directly affects granulosa cells and coordinates with GDF9 and BMP15 to regulate gene expression in preantral follicles. OOX‐PAGCs were cultured in a medium supplemented with or without 50 ng mL^−1^ recombinant RSPO2, 30 ng mL^−1^ recombinant mouse GDF9:BMP15 heterodimer, and a combination of RSPO2 (50 ng mL^−1^) + GDF9:BMP15 (30 ng mL^−1^) for 48 h. RNA‐seq analysis was then performed to assess changes in their transcriptome (**Figure** **2**A; Figure S3, Supporting Information).

The results demonstrated that RSPO2 treatment induced the differential expression of 3012 genes in OOX‐PAGCs, with 1417 genes upregulated and 1595 downregulated by RSPO2 (Figure 2B; Figure S4A, Table S3, Supporting Information). Gene enrichment analysis revealed that genes upregulated by RSPO2 were primarily involved in anabolic processes, particularly the “small molecule biosynthetic process,” “glycolytic process,” “cholesterol biosynthesis,” and “nucleotide biosynthetic process” (Figure S4B, Supporting Information). Additionally, genes upregulated by RSPO2 were associated with the “negative regulation of the catabolic process,” all of which are crucial for cell proliferation and survival. In contrast, genes suppressed by RSPO2 were linked to “vasculature development,” “actin filament‐based processes,” “extracellular matrix organization,” and “negative regulation of cell proliferation/differentiation/locomotion,” which are not typical of PAGCs (Figure S4C, Supporting Information).

Treatment with the GDF9:BMP15 heterodimer or the combination of RSPO2 + GDF9:BMP15 induced changes in the expression of 6359 genes (3482 upregulated and 2877 downregulated) and 6451 genes (3540 upregulated and 2911 downregulated), respectively, in OOX‐PAGCs (Figure 2B; Figure S4D,G, Table S3, Supporting Information). Interestingly, genes regulated by these two treatments were involved in similar biological processes. Specifically, genes upregulated by both treatments were primarily associated with “cell cycle,” “RNA metabolism,” and “cholesterol biosynthesis,” while genes downregulated by both treatments were linked to “actin filament‐based processes,” “extracellular matrix organization,” and “vasculature development” (Figure S4E–I, Supporting Information).

**Figure 3:**
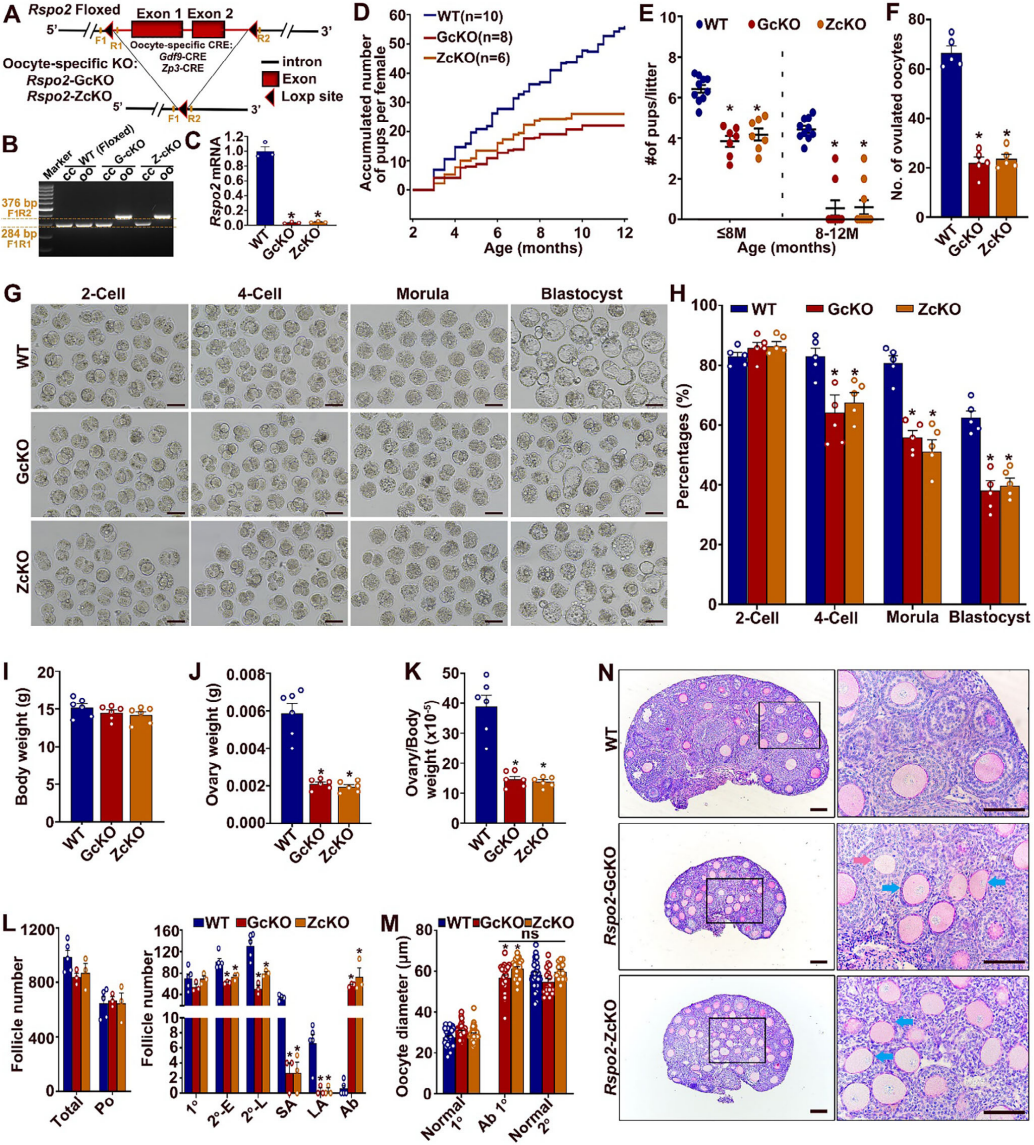
Oocyte‐specific knockout of *Rspo2* in mice compromises oocyte and follicle development, leading to subfertility and earlier termination of reproductive lifespan. A) Schematic representation of the experimental design for oocyte‐specific knockout (KO) of the *Rspo2* gene in mice. B) Representative DNA electrophoresis gel showing PCR genotyping results for oocyte‐specific deletion of the floxed DNA fragment in *Rspo2*‐GcKO females and *Rspo2*‐ZcKO females. Genomic DNA was extracted from the oocytes (OO) and cumulus cells (CC) of *Rspo2* ^fl/fl^ (WT (Floxed)), and *Rspo2*‐cKO (G‐cKO, Z‐cKO) mice, and amplified using primers F1 with R1 or R2 as indicated in (A), yielding a 284‐bp product for the floxed allele and a 376‐bp product for the null allele. The predicited 2685‐bp WT product was not successfuly amplified due to inefficient amplification of long framents. C) Bar graph showing the qRT‐PCR analysis of *Rspo2* mRNA levels in oocytes from *Rspo2*‐cKO (G‐cKO, Z‐cKO) mice, confirming the knockout efficiency. Data are presented as Mean ± SEM (*N* = 3). ^*^ *p*<0.05, cKO versus WT. D, E) Fertility assessment of WT (*N* = 10), *Rspo2*‐GcKO (GcKO, *N* = 8), and *Rspo2*‐ZcKO (ZcKO, *N* = 6) female mice: (D) shows the cumulative number of pups per female from 2 to 12 months of age; (E) presentss the number of pups per female during 2–8 and 8–12 months. Data are shown as Mean ± SEM. ^*^ *p*<0.05, cKO versus WT. F) Bar graph showing the difference in the number of ovulated oocytes between WT and *Rspo2*‐cKO females. Data are presented as Mean ± SEM (*N* = 5). ^*^ *p*<0.05, cKO versus WT. G, H) Representative micrographs (G) and developmental rate (H) of 2‐cell, 4‐cell, morula, and blastocyst‐stage embryos formed by the ovulated WT, *Rspo2*‐GcKO and ZcKO oocytes after IVF. Scale bars represent 100 µm. Data are presented as Mean ± SEM (*N* = 5). ^*^ *p*<0.05, *Rspo2*‐cKO versus the WT. I–K) Comparison of body weight (I), ovarian weight (J), and ovarian‐to‐body weight ratio (K) between the 21‐day‐old WT and *Rspo2‐*cKO females. Data are presented as Mean ± SEM (*N* = 6). ^*^ *p*<0.05, *Rspo2*‐cKO versus the WT. L) Quantification of the number of follicles at different stages of development in the 21‐day‐old WT, *Rspo2*‐GcKO, and *Rspo2*‐ZcKO females. Follicle stages are indicated as: Po, primordial follicles; 1°, primary follicles; 2°‐E, early secondary follicles; 2°‐L, late secondary follicles; SA, small antral follicles; LA, large antral follicles; Ab, abnormal follicles. Data are presented as Mean ± SEM (WT, *N* = 5; *Rspo2*‐GcKO and *Rspo2*‐ZcKO, *N* = 3). ^*^ *p*<0.05, *Rspo2*‐cKO versus WT. M) Diameters of oocytes in normal primary (1°) and secondary (2°) follicles from WT and *Rspo2*‐cKO mice, as well as abnormal primary follicles from *Rspo2*‐cKO mice. Data are presented as Mean ± SEM (normal 1°, *N* = 30; abnormal 1°, *N* = 20; normal 2°, *N* = 15). ^*^ *p*<0.05, abnormal 1° versus WT normal 1°. N) Micrographs of PAS‐stained ovarian sections from 21‐day‐old WT, *Rspo2*‐GcKO, and *Rspo2*‐ZcKO mice. Enlarged views of the boxed area are shown on the right. Blue arrows indicate follicles with a single‐layer or no granulosa cells, while red arrows point to follicles with loosely packed granulosa cells. Nuclei are counterstained with hematoxylin. Scale bars = 100 µm.

**Figure 4:**
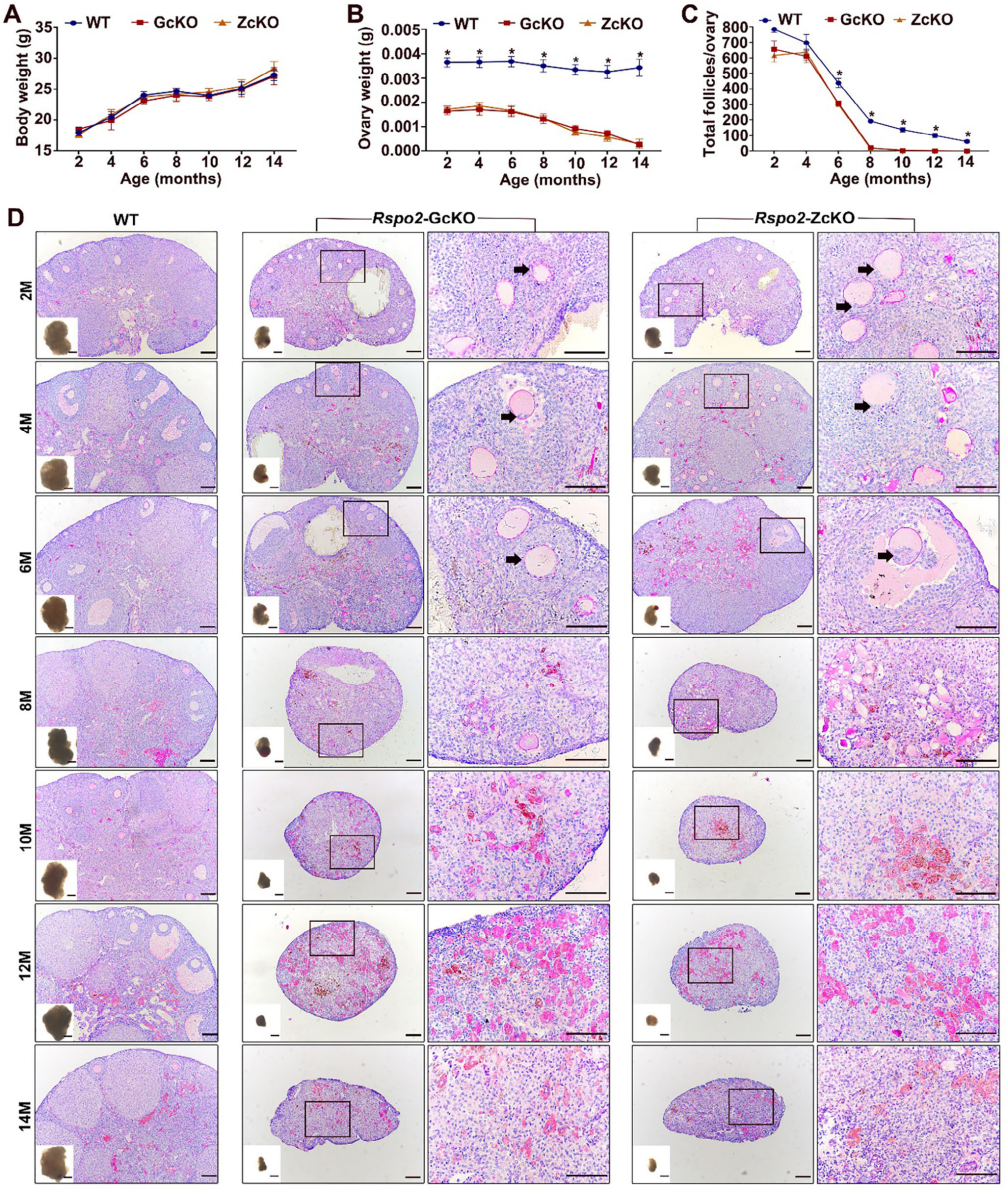
Age‐dependent deterioration of follicular defects and premature depletion of ovarian follicles in *Rspo2*‐cKO females. A–C) Age‐dependent changes in body weight (A), ovarian weight (B), and total follicle count per ovary (C) in WT and *Rspo2*‐cKO females. Data are presented as Mean ± SEM (*N* = 3). ^*^ *p*<0.05, *Rspo2*‐cKO versus WT at the same age. D) Representative micrographs of ovarian sections at various ages, stained with PAS and hematoxylin. The inset shows the morphology of the ovary before sectioning. Enlarged views of the boxed area are shown on the right, with arrows indicating oocytes with granulosa cells infiltrating the cytoplasm. Scale bars = 100 µm.

Further analysis revealed that 1335 (40+ 1068+ 32+ 195), 2280 (1068+ 1116+ 64+ 32), and 2270 (40+ 1068+ 1116+ 46) genes regulated by RSPO2, GDF9:BMP15, and RSPO2+GDF9:BMP15, respectively, were also regulated by oocytes and OSFs (Oo‐OSFs), accounting for 43.5%, 74.2%, and 73.9% of the Oo‐OSF‐regulated genes (Figure 2B). Intersecting the datasets of genes regulated by RSPO2 and GDF9:BMP15, we identified 2193 (80+ 1013+ 1068+ 32) genes commonly regulated by both factors, constituting 72.8% of the RSPO2‐regulated genes (Figure 2B). These genes were further categorized into four distinct groups based on their mode of regulation: 1) Genes promoted by both RSPO2 and GDF9:BMP15 (537) (Figure 2C); 2) Genes suppressed by both RSPO2 and GDF9:BMP15 (889) (Figure 2D); 3) Genes promoted by RSPO2 but suppressed by GDF9:BMP15 (406) (Figure 2E); 4) Genes suppressed by RSPO2 but promoted by GDF9:BMP15 (361) (Figure 2F).

Gene enrichment analysis of the first category revealed that these genes were primarily involved in metabolic processes (e.g., “small molecule biosynthetic process,” “nucleoside diphosphate metabolic process,” “lipid/fatty acid/cholesterol biosynthesis”), cell division (e.g., “mitotic prometaphase,” “mitotic spindle organization”), and cellular responses to hypoxia and stress (Figure 2C). Notably, genes in this category include those crucial for granulosa cell, oocyte, and/or follicle development, such as *Amh*, *Ar*, *Ccnd2*, *Cyp19a1*, *Dhh, Esr1*, *Fst*, *Fshr*, *Grem2*, *Inha*, *Inhbb*, *Npr2*, and *Wnt5b* (Figure 2G). These changes were further validated by qRT‐PCR (Figure 2K).

Genes suppressed by both RSPO2 and GDF9:BMP15 (Category 2) were involved in processes similar to those suppressed by Oo‐OSFs, including “actin filament‐based processes” (e.g., *Cav2*, *Cxcl12*, and *Fgd5*), “enzyme‐linked receptor protein signaling pathway” (e.g., *Ghr*, *Irs2*, and *Pik3cd*), “vasculature development” (e.g., *Angpt2*, *Sema5a*, and *Thbs1*), and the “negative regulation of cell population proliferation”(e.g., *Pawr*, *Sox4*, and *Thbs1*) (Figure 2D,H). Notably, genes encoding components of the BMP signaling pathway (i.e., *Bmp2*, *Bmpr2*, *Smad6*, and *Smad9*) and negative regulators of the WNT/CTNNB pathway (i.e., *Dact1* and *Dkkl1*) were also within this category. These changes were further confirmed by qRT‐PCR (Figure 2H,J).

Most intriguingly, in addition to the aforementioned genes regulated by RSPO2 and GDF9:BMP15 in a synergistic manner, we identified 767 genes whose regulation by RSPO2 was antagonistic to that of GDF9:BMP15 (Figure 2E,F). Among these, 406 genes were promoted by RSPO2 but suppressed by GDF9:BMP15 (Category 3) (Figure 2E), and 361 genes were suppressed by RSPO2 but promoted by GDF9:BMP15 (Category 4) (Figure 2F). These genes were associated with distinct biological processes and pathways. Changes in the expression of representative genes from these categories are illustrated in the heatmaps of Figure 2I,J. Notably, genes crucial for granulosa cell and/or oocyte development, such as *Amhr2*, *Kitl*, and *Smad3*, as well as *Cldn11* — a key tight junction component expressed in granulosa cells upon transdifferentiated into Sertoli cells — were promoted by RSPO2 but suppressed by GDF9:BMP15 (Figure 2M). Additionally, members of the WNT family, including *Wnt1* and *Wnt 6*, were also promoted by RSPO2 but suppressed by GDF9:BMP15 (Figure 2M). In contrast, genes associated with BMP signaling (e.g., *Acvr1*, *Bmpr1b*, and *Smad7*), transcription factors important for granulosa cell development (e.g., *Fos* and *Myc*), as well as gap junction proteins and amino acid transporters highly expressed in granulosa cells (e.g., *Gja1* and *Slc38a3*), were suppressed by RSPO2 but promoted by GDF9:BMP15 (Figure 2N).

### 2.3 Conditional Knockout of *Rspo2* in Oocytes Causes Defective Oocyte and Follicle Development, Leading to Earlier Termination of the Reproductive Lifespan

To explore the potential role of RSPO2 in oocyte and follicle development under physiological conditions, we conditionally knocked out *Rspo2* in oocytes using *Gdf9*‐Cre and *Zp3*‐Cre mice, where CRE recombinase is specifically expressed in oocytes from the non‐growing primordial follicle and growing primary follicle stages onward, respectively (**Figure** **3**A). These two oocyte‐specific *Rspo2*‐conditional knockout models are referred to as *Rspo2*‐GcKO and *Rspo2*‐ZcKO, respectively. Genotyping PCR using primers flanking the floxed region confirmed the specific deletion of the targeted DNA sequence in oocytes, but not in cumulus cells, of both *Rspo2*‐GcKO and *Rspo2*‐ZcKO female mice (Figure 3B). Quantitative RT‐PCR analysis revealed that *Rspo2* mRNA was nearly undetectable in oocytes of both *Rspo2*‐cKO female models (Figure 3C).

Fertility assays revealed that both *Rspo2*‐cKO female groups exhibited significantly reduced fecundity (Figure 3D). By 8 months of age, the *Rspo2*‐GcKO and *Rspo2*‐ZcKO mice produced an average of only 4.11 and 4.18 pups per mouse, respectively, compared to 6.43 pups per mouse in WT controls (Figure 3E). More notably, the *Rspo2*‐cKO females became nearly sterile after 8 months of age, with no pups produced at 10 months and beyond, while their WT littermate controls remained fertile at the same age, producing an average litter size of 4.44 (Figure 3E).

To investigate the mechanisms underlying the reduced fertility in the *Rspo2*‐cKO female mice, we assessed the ovulation capacity of both *Rspo2*‐cKO groups through a superovulation experiment and examined the developmental competence of the ovulated oocytes using in vitro fertilization (IVF) and embryo culture. The results indicated that both *Rspo2*‐cKO groups ovulated fewer oocytes compared to WT controls (Figure 3F). Additionally, while the *Rspo2*‐cKO oocytes were fertilized normally by WT sperm in vitro and formed 2‐cell embryos at a rate similar to WT oocytes, further development of the 2‐cell embryos derived from *Rspo2*‐cKO oocytes was significantly compromised (Figure 3G,H). These results suggest that a decline in both the number and quality of the ovulated oocytes is a key contributor to the reduced fertility observed in the *Rspo2*‐cKO females.

The reduction in ovulated oocytes prompted us to examine the possibility that follicular development might be impaired in the *Rspo2*‐cKO mice. We assessed follicular development in 21‐day‐old prepubertal females and found that although both *Rspo2*‐cKO groups had body weights comparable to WT mice (Figure 3I), the ovaries of the *Rspo2*‐cKO mice were much smaller (Figure 3J), with the ovarian‐to‐body weight ratio reduced to approximately one‐third of that in WT mice (Figure 3K; Figure S5A, Supporting Information). Follicle counts revealed no significant difference in the total number of follicles, nor in the numbers of primordial or primary follicles between WT and *Rspo2*‐cKO mice (Figure 3L). However, the number of secondary follicles and more advanced follicles was significantly reduced in both *Rspo2*‐cKO groups (Figure 3L). Notably, many of the remaining follicles exhibited abnormal morphology in both *Rspo2*‐GcKO and *Rspo2*‐ZcKO ovaries. These abnormal follicles contained oocytes that were larger than those in normal primary follicles, and their granulosa cells displayed various defects (Figure 3M,N). The majority (86% in *Rspo2*‐GcKO and 91% in *Rspo2*‐ZcKO) of these abnormal follicles had only a single layer of flattened granulosa cells or almost no granulosa cells surrounding the oocyte, while the remaining follicles contained irregularly packed granulosa cells around the oocyte (Figure 3N; Figure S5H,I, Supporting Information). A few follicles even showed granulosa cells invading the oocyte (Figure S5H,I, Supporting Information). Similar follicular defects were observed after priming the mice with eCG or eCG+hCG to stimulate antral follicle development and ovulation (Figure S5, Supporting Information). Interestingly, although fewer FGOs were retrieved from the *Rspo2*‐cKO mice after eCG priming, a higher proportion of these retrieved FGOs contained oocytes with granulosa cell invasion (Figure S5C,D, Supporting Information). After hCG administration, a large proportion of periovulatory follicles in the *Rspo2*‐cKO mice showed a reduction in the number of cumulus cells surrounding the oocyte (Figure S5F,G, Supporting Information). Additionally, following eCG or eCG+hCG priming, more abnormal follicles containing irregularly arranged granulosa cells around the oocyte were observed in the *Rspo2*‐cKO mice (Figures S5H,J,K, Supporting Information). Collectively, these data indicate that defective follicular development is a major contributor to the reduced number of ovulated oocytes in the *Rspo2*‐GcKO and *Rspo2*‐ZcKO mice.

### 2.4 Age‐Dependent Deterioration of Follicular Defects and Earlier Ovarian Follicle Depletion in *Rspo2*‐cKO Females

To investigate the underlying causes of the earlier termination of reproductive lifespan in *Rspo2*‐cKO females, we first examined whether there were defects in the establishment of the ovarian follicle pool. We assessed the number of primordial follicles by immunofluorescence (IF) staining ovarian sections from 3‐day‐old WT and *Rspo2*‐GcKO mice with DDX4 and FOXL2 antibodies to label oocytes and pregranulosa cells. The results indicated that primordial follicles were assembled normally within the ovaries of 3‐day‐old *Rspo2*‐GcKO mice (Figure S6A, Supporting Information), and their number did not differ from that of WT mice (Figure S6B, Supporting Information). We then examined age‐dependent changes in ovarian follicle development in adult mice. The results revealed that although both *Rspo2*‐GcKO and *Rspo2*‐ZcKO mice grew normally and maintained body weights comparable to WT controls (**Figure** **4**A), their ovaries were significantly smaller at all ages examined, as assessed by ovarian weight (Figure 4B). Notably, dramatic ovarian shrinkage occurred in the *Rspo2*‐cKO mice after 8 months of age (Figure 4B,D). Follicle count analysis showed that the total number of follicles in *Rspo2*‐cKO mice was significantly lower than that in WT mice by 6 months of age and beyond (Figure 4C,D). An abrupt decline in the total follicle count occurred when the *Rspo2*‐cKO mice reached 8 months, followed by complete depletion of all ovarian follicles by 10 months of age (Figure 4C,D). The most notable feature of the abnormal follicles in the ovaries of 2‐6‐month‐old *Rspo2*‐cKO mice was the invasion of granulosa cells into the oocyte (Figure 4D). These data collectively indicate that the premature depletion of ovarian follicles, rather than defective establishment of the follicle pool, is the primary cause of the earlier termination of reproductive lifespan in the *Rspo2*‐cKO mice.

### 2.5 Impaired Proliferation, Survival, and TZP Formation in Granulosa Cells of *Rspo2*‐cKO Mice

**Figure 5:**
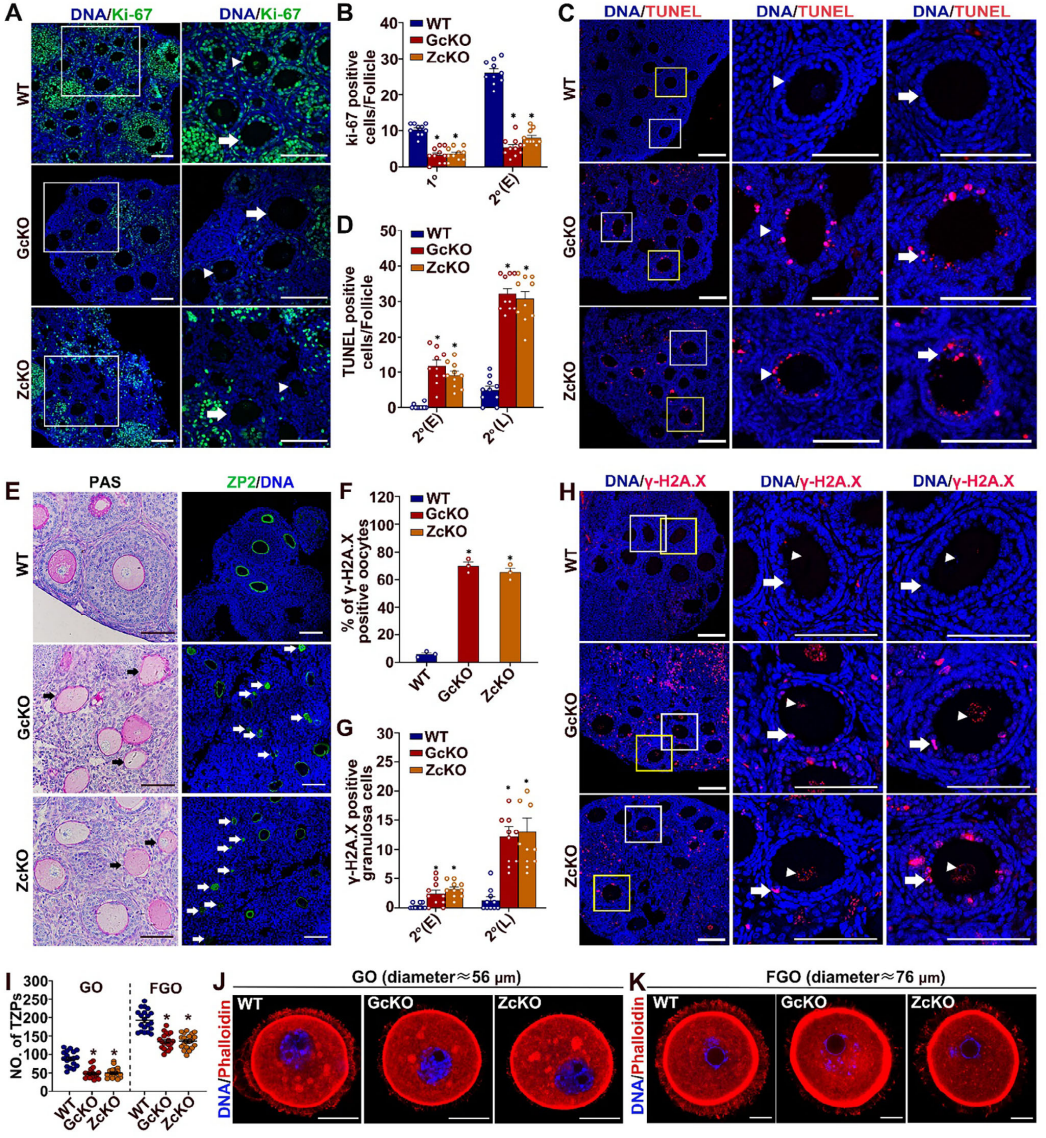
Impaired proliferation, survival, and TZP formation in granulosa cells of *Rspo2*‐cKO mice. A) Immunofluorescence (IF) staining for Ki‐67 (green) on the ovarian section from 21‐day‐old wild‐type (WT), *Rspo2*‐GcKO (GcKO), and *Rspo2*‐ZcKO (ZcKO) females. Enlarged views of the boxed area are shown on the right, with arrowheads and arrows indicating primary and early secondary follicles, respectively. DNA is counterstained with DAPI (blue). Scale bars = 100 µm. B) Quantification of the number of Ki‐67‐positive granulosa cells in primary (1°) and early secondary (2°) follicles. A total number of 10 primary and 10 secondary follicles were counted for each genotype. Data are presented as Mean ± SEM. ^*^ *p*<0.05, *Rspo2*‐cKO versus WT. C) TUNEL staining (red) of ovarian section from 21‐day‐old wild‐type (WT), *Rspo2*‐GcKO (GcKO), and *Rspo2*‐ZcKO (ZcKO) females. Amplified views of the boxed area are shown on the right, with arrowheads and arrows pointing to early and late secondary follicles, respectively. DNA is counterstained with DAPI (blue). Scale bars = 100 µm. D) Quantification of TUNEL‐positive granulosa cells in early (2°E) and late (2°L) secondary follicles. A total number of 10 early secondary and 10 late secondary follicles were counted for each genotype. Data are presented as Mean ± SEM. ^*^ *p*<0.05, *Rspo2*‐cKO versus WT. E) Micrographs of ovarian sections from 21‐day‐old female mice, stained with PAS (left panel) or for ZP2 by IF (right panel), showing follicles with degenerated oocytes, as indicated by arrows. Nuclei were counterstained with hematoxylin, and DNA with DAPI (blue). Scale bars = 100 µm. F–H) Analysis of DNA double‐strand breaks in the oocyte and granulosa cells by IF staining of γ‐H2A.X: (F) Quantification of the IF staining results in oocytes, with a total of 30 oocytes counted for each genotype; (G) Quantification of the IF staining results in granulosa cells, with 10 early secondary and 10 late secondary follicles counted for each genotype; (H) Representative micrographs of the IF staining, with γ‐H2A.X shown in red and DNA counterstained with DAPI (blue). Enlarged views of the boxed area are shown on the right, with arrowheads and arrows indicating the oocytes and granulosa cells, respectively. Scale bars = 100 µm. Data are presented as Mean ± SEM. ^*^ *p*<0.05, *Rspo2*‐cKO versus WT. I–K) Assessment of TZPs in *Rspo2*‐cKO oocytes: (I) Quantification of the number of TZPs per growing oocyte (GO) and fully‐growing oocyte (FGO), with 18 GOs and 20 FGOs counted for each genotype. Data are presented as Mean ± SEM. ^*^ *p*<0.05, *Rspo2*‐cKO versus WT; (J) Micrographs showing the Phalloidin‐stained TZPs (red) in GOs; (K) Micrographs showing the Phalloidin‐stained TZPs (red) in FGO. Scale bars indicate 20 µm.

To investigate the underlying causes of defective follicular development in *Rspo2*‐cKO mice, we assessed cell proliferation and apoptosis during folliculogenesis in the ovaries of 21‐day‐old prepubertal females using Ki‐67 and TUNEL staining, respectively. We observed significant differences in cell proliferation, with fewer Ki‐67‐positive granulosa cells in the primary and early secondary follicles of *Rspo2*‐cKO mice (**Figure** **5**A,B). Conversely, there was an increased number of TUNEL‐positive granulosa cells in both early and late secondary follicles of *Rspo2*‐GcKO and *Rspo2*‐ZcKO ovaries (Figure 5C,D).

Corresponding to the heightened apoptosis, histological analysis following Periodic Acid‐Schiff (PAS) staining revealed that many early‐stage growing follicles in the *Rspo2*‐cKO mice contained degenerated oocytes. These oocytes lacked an intact zona pellucida, as evidenced by IF staining of ZP2, where the zona pellucida appeared to collapse into clumps (Figure 5E). Oocyte degeneration and death in early‐stage growing follicles are often linked to the accumulation of excessive DNA damage. To test this, we performed IF staining for γH2AX, a marker for DNA double‐strand breaks (DSBs), on ovarian sections from 21‐day‐old mice. The results showed an increased number of DSBs in both *Rspo2*‐cKO oocytes and granulosa cells of early‐stage growing follicles (Figure 5F–H).

Moreover, the abnormal development of granulosa cells, oocytes, and follicles observed in *Rspo2*‐cKO mice may result from defective interactions between the oocyte and granulosa cells. To investigate this, we assessed TZPs and oocyte microvilli, the structural basis for oocyte‐granulosa cells communication, by labeling actin filaments with rhodamine‐conjugated Phalloidin and performing IF staining of Radixin (RDX) and its activated form, phosphorylated (p‐) ERM — key components of the oocyte microvilli. The results showed that the number of TZPs within the zona pellucida of growing oocytes and FGOs was significantly reduced in the *Rspo2*‐cKO mice (Figure 5I–K). However, the expression levels of RDX and p‐ERM, as well as the number of microvilli, were not altered in the *Rspo2*‐cKO oocytes (Figure S6C–J, Supporting Information).

These data indicate that *Rspo2* deletion in oocytes impairs the proliferation and survival of granulosa cells, as well as the structural basis of oocyte‐granulosa cell communication. This disruption likely contributes to defective follicular development, resulting in the progressive accumulation of DNA damage in developing oocytes, leading to their eventual loss and the premature depletion of follicles with age.

### 2.6 Loss of Transcriptomic Integrity in Granulosa Cells and Oocytes of *Rspo2*‐cKO Mice

**Figure 6:**
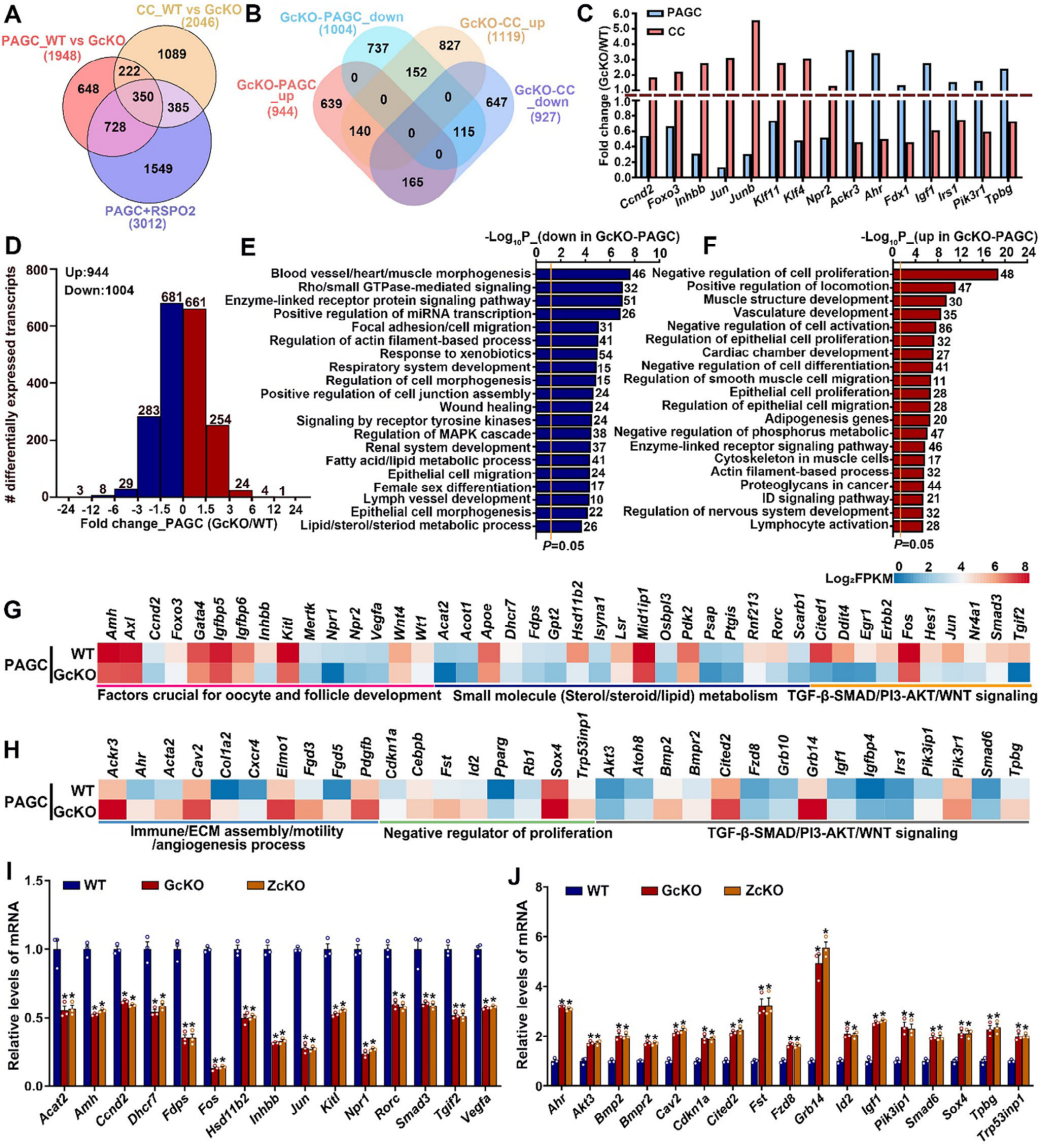
Loss of transcriptomic integrity in granulosa cells of *Rspo2* oocyte‐specific knockout mice. A) Venn diagram comparing DEGs in preantral follicle granulosa cells (PAGCs) and antral follicle cumulus cells (CCs) from *Rspo2*‐GcKO (GcKO) mice with DEGs in WT‐PAGCs following RSPO2 treatment. The number of DEGs in each group is shown in the parentheses. B) Venn diagram comparing the upregulated and downregulated DEGs in PAGCs and CCs from *Rspo2*‐GcKO (GcKO) mice. The number of DEGs in each group is indicated in parentheses. C) Fold changes of representative DEGs showing opposite directional changes in PAGCs and CCs from *Rspo2*‐GcKO (GcKO) mice. D) Distribution of DEGs at various magnitudes of expression level differences between WT and *Rspo2*‐GcKO PAGCs, as detected by RNA‐Seq. The number of genes in each category is indicated above each bar. E, F) Bar graphs showing the pathways and biological processes associated with downregulated (E) and upregulated (F) genes in *Rspo2*‐GcKO PAGCs. G, H) Heatmaps showing expression changes of selected genes in *Rspo2*‐GcKO PAGCs that are essential for granulosa cell, oocyte, and follicle development, with downregulated genes displayed in (G) and the the upregulated genes in (H). I, J) qRT‐PCR validation of changes in representative gene expression selected from the downregulated (I) and upregulated (J) DEGs in *Rspo2*‐GcKO PAGCs. Data are presented as Mean ± SEM (*N* = 3). ^*^ *p*<0.05, *Rspo2*‐cKO versus WT.

To explore the molecular mechanisms by which specific depletion of RSPO2 in oocytes affects granulosa cell development and contributes to defective oocyte and follicle development in *Rspo2*‐cKO mice, we first compared the transcriptomes of granulosa cells between WT and *Rspo2*‐GcKO mice. Since previous experimental results showed no significant difference between the *Rspo2*‐GcKO and *Rspo2*‐ZcKO mice, the following granulosa cell and oocyte transcriptome experiments were carried out only on the *Rspo2*‐GcKO mice. RNA‐Seq analysis identified 1948 differentially expressed genes (DEGs) in PAGCs and 2046 DEGs in cumulus cells of *Rspo2*‐GcKO mice (**Figure** **6**A; Tables S4 and S5, Supporting Information). When we intersected these DEGs with the dataset of RSPO2‐regulated genes from cultured normal WT PAGCs, we found that 1078 (728+ 350) DEGs in *Rspo2*‐GcKO PAGCs and 735 (350+ 385) DEGs in *Rspo2*‐GcKO cumulus cells were also directly regulated by RSPO2 in WT PAGCs (Figure 6A).

Interestingly, only 29.4% (572 out of 1948 total DEGs) of the DEGs in PAGCs overlapped with those in cumulus cells of *Rspo2*‐GcKO mice (Figure 6A). More notably, among the 572 (165+ 115+ 152+ 140) genes that were differentially expressed in both PAGCs and cumulus cells, 317 (165+ 152) were found to be changed in opposite directions in these two cell populations (Figure 6B). These results suggest that RSPO2, secreted by the oocyte, may have distinct effects in preantral versus antral follicles by regulating different subsets of genes in the surrounding granulosa cells. The differential expression of representative genes in PAGCs and cumulus cells is shown in Figure 6C. These include genes that were downregulated in PAGCs but upregulated in cumulus cells (e.g., *Ccnd2*, *Foxo3*, *Inhb* *b*, *Jun*, *Junb*, *Klf1*, *Kif4*, and *Npr2*), as well as those that were upregulated in PAGCs but downregulated in cumulus cells (e.g., *Ackr3*, *Ahr*, *Fdx1*, *I* *gf1*, *Irs1*, *Prk3r1*, and *Tpbg*).

Of the 1948 genes differentially expressed in PAGCs of *Rspo2*‐GcKO mice, 1004 were downregulated, and 944 were upregulated (Figure 6D; Table S4, Supporting Information). Gene enrichment analysis of these DEGs revealed associated biological pathways and processes, shown in Figure 6E,F. Notably, the “fatty acid/lipid metabolic process” and “lipid/sterol/steroid metabolic process”—critical for oocyte and granulosa cell development—were among the pathways associated with the downregulated genes in PAGCs of *Rspo2*‐GcKO mice (Figure 6E). In contrast, the most prominent biological pathways and processes associated with the upregulated genes included “negative regulation of cell proliferation/differentiation,” “angiogenesis/motility/immune process,” and “ECM assembly” (Figure 6F). Changes in the expression of genes involved in these processes (e.g., *Dhcr7* and *Fdps* in “sterol biosynthesis”; *Ahr*, *Col1a2*, *Elmo1*, and *Pdgfb* in “immune/ECM assembly/motility/angiogenesis”; *Cdkn1a* and *Fst* in “negative regulation of cell proliferation”) may, at least in part, contribute to the defective development of granulosa cells, oocytes, and follicles in *Rspo2*‐GcKO mice (Figure 6G,H).

Consistent with this hypothesis, a detailed analysis of genes in these pathways revealed several factors crucial for oocyte and follicle development, such as *Amh*, *Ccnd2*, *Inhbb*, *Kitl*, *Npr2*, *Wnt4*, and *Wt1*, which were downregulated in *Rspo2*‐GcKO PAGCs (Figure 6G,I). Interestingly, some genes involved in the “TGF‐β‐SMAD/PI3‐AKT/WNT signaling” pathway, including *Ddit4*, *Hes* *1*, and *Smad3*, were downregulated (Figure 6G,I), while others, such as *Fzd8*, *Pik3ip1*, and *Smad6*, were upregulated in *Rspo2*‐GcKO PAGCs (Figure 6H,J). This highlights the complex interplay between RSPO2 signaling and other important pathways in PAGCs. Changes in the expression of these key genes were illustrated by heatmap and further validated by qRT‐PCR (Figure 6G–J).

**Figure 7:**
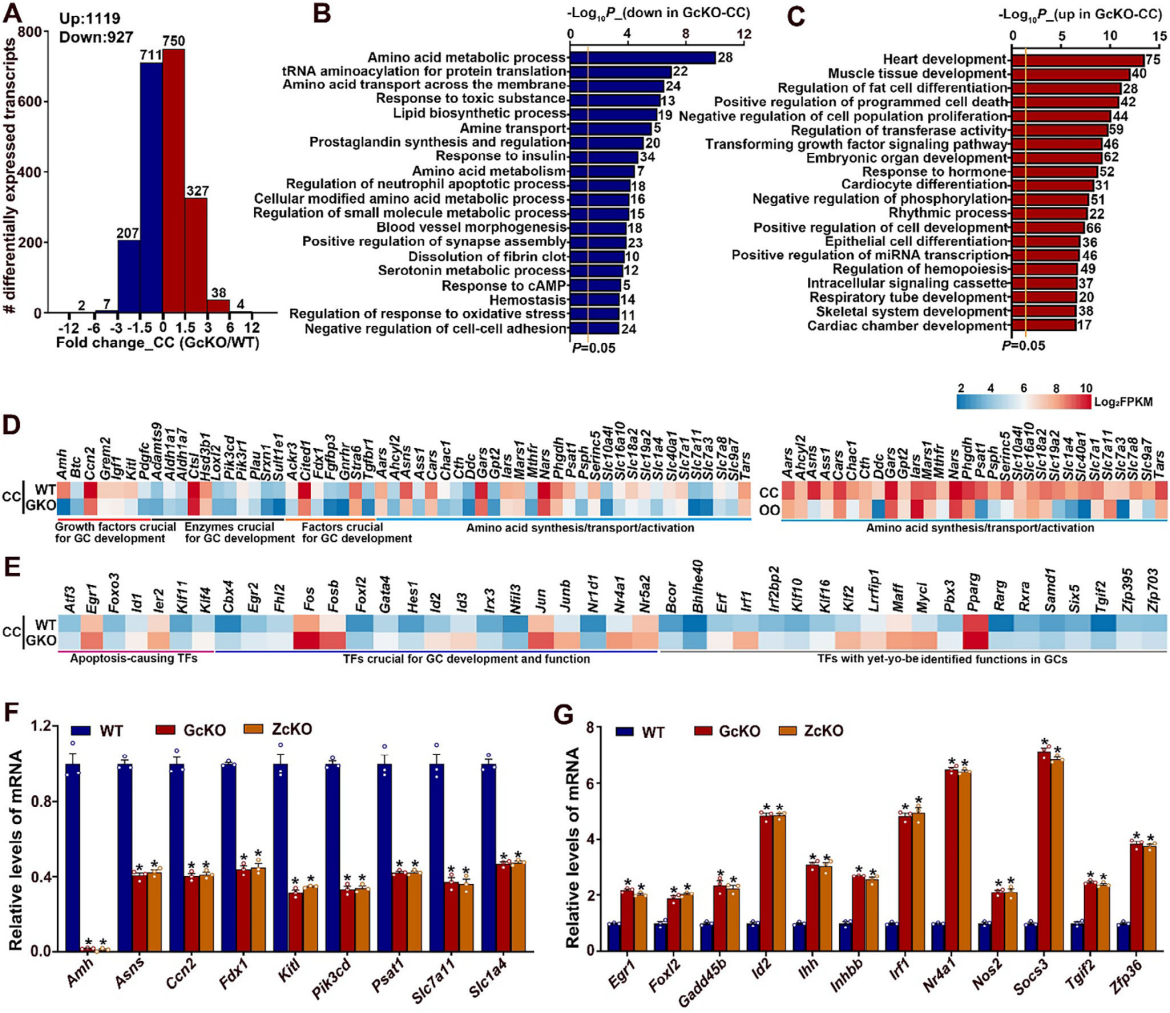
Distortion of the cumulus cell transcriptome in *Rspo2* oocyte‐specific knockout mice. A) Distribution of DEGs at various magnitudes of expression level differences between WT and *Rspo2*‐GcKO cumulus cells (CCs) detected by RNA‐Seq. The number of genes in each category is indicated above each bar. B,C) Bar graphs depicting the pathways and biological processes associated with downregulated (B) and upregulated (C) genes in *Rspo2*‐GcKO CCs. D, E) Heatmaps showing expression changes of selected genes in *Rspo2*‐GcKO CCs that are essential for granulosa cell, oocyte, and follicle development, with downregulated genes displayed in (D) and the the upregulated genes in (E). The right panel in (D) highlights the differential expression of genes involved in “amino acid metabolism/transport/activation” in CCs compared to oocytes. F, G) qRT‐PCR validation of changes in representative gene expression selected from the downregulated (F) and upregulated (G) DEGs in *Rspo2*‐GcKO CCs. Data are presented as Mean ± SEM (*N* = 3). ^*^ *p*<0.05, *Rspo2*‐cKO versus WT.

A total of 2046 genes were identified as differentially expressed in *Rspo2*‐GcKO cumulus cells, with 927 downregulated and 1119 upregulated (**Figure** **7**A; Table S5, Supporting Information). Gene enrichment analysis revealed that the downregulated genes were predominantly associated with metabolic processes, particularly those related to “amino acid metabolism/transport/activation,” which are critical for cell development and survival (Figure 7B). Notably, a majority of the genes involved in these processes (27 out of 30, including *Asns*, *Psat1*, and *Slc1a4*) were expressed at significantly higher levels in cumulus cells compared to oocytes (Figure 7D,F). This suggests a potential cooperative relationship between cumulus cells and oocytes in regulating “amino acid metabolism/transport/activation,” highlighting their interdependent roles in metabolic processes essential for folliculogenesis.

Additionally, several genes crucial for granulosa/cumulus cell and oocyte development, including growth factors (e.g., *Amh*, *Ccn2/Ctgf*, *Grem2*, *Kitl*, and *Igf1*), enzymes (e.g., *Adamts9*, *Ctsl*, *Pik3cd*, and *Plau*), and other factors (e.g., *Aldh1a7*, *Fdx1*, *Gnrhr*, and *Tgfbr1*), were downregulated in *Rspo2*‐GcKO cumulus cells (Figure 7D,F). For the 1119 genes upregulated in *Rspo2*‐GcKO cumulus cells, gene enrichment analysis revealed involvement in organ development (e.g., heart, muscle, respiratory tube, and skeletal system), cell differentiation (e.g., fat, cardiocyte, and epithelial cells), and processes like “positive regulation of programmed cell death” and “negative regulation of cell population proliferation” (Figure 7C). These findings indicate maldevelopment and dysfunctional behavior of cumulus cells in the *Rspo2*‐cKO mice.

Interestingly, a substantial number of genes encoding transcriptional factors and regulators were upregulated in the *Rspo2*‐cKO cumulus cells (Figure 7E). These included transcription factors known to induce apoptosis (e.g., *Atf3*, *Egr1*, *Foxo3*, *Id1*, *Ier2*, *Kif11*, and *Kif4*), those crucial for granulosa cell development and function (e.g., *Cbx4*, *Egr2*, *Fhl2*, *Fos*, *Fos* *b*, *Foxl2*, *Gata4*, *Hes1*, *Id2*, *Id3*, *Irx3*, *Jun*, *Junb*, *Nfil3*, *Nr1d1*, *Nr4a1*, and *Nr5a2*), and some with potential but yet‐to‐be identified roles in granulosa cells (e.g., *Pparg*, *Rarg*, and *Tgif2*). Notably, the majority of genes in the latter category (13 out of 20) encode transcriptional repressors. Changes in the expression of representative genes from these transcriptional factors (e.g., *Egr1*, *Foxl2*, *Id2*, *Irf1*, *Nr4a1*, and *Tgif2*), as well as genes critical for granulosa cell development and survival (e.g., *Gadd45b*, *Inhbb*, *Nos2*, and *Socs2*), theca cell development (e.g., *Ihh*), and the reinitiation of oocyte meiosis after the preovulatory LH surge (e.g., *Zfp36*), were validated by qRT‐PCR (Figure 7G).

**Figure 8:**
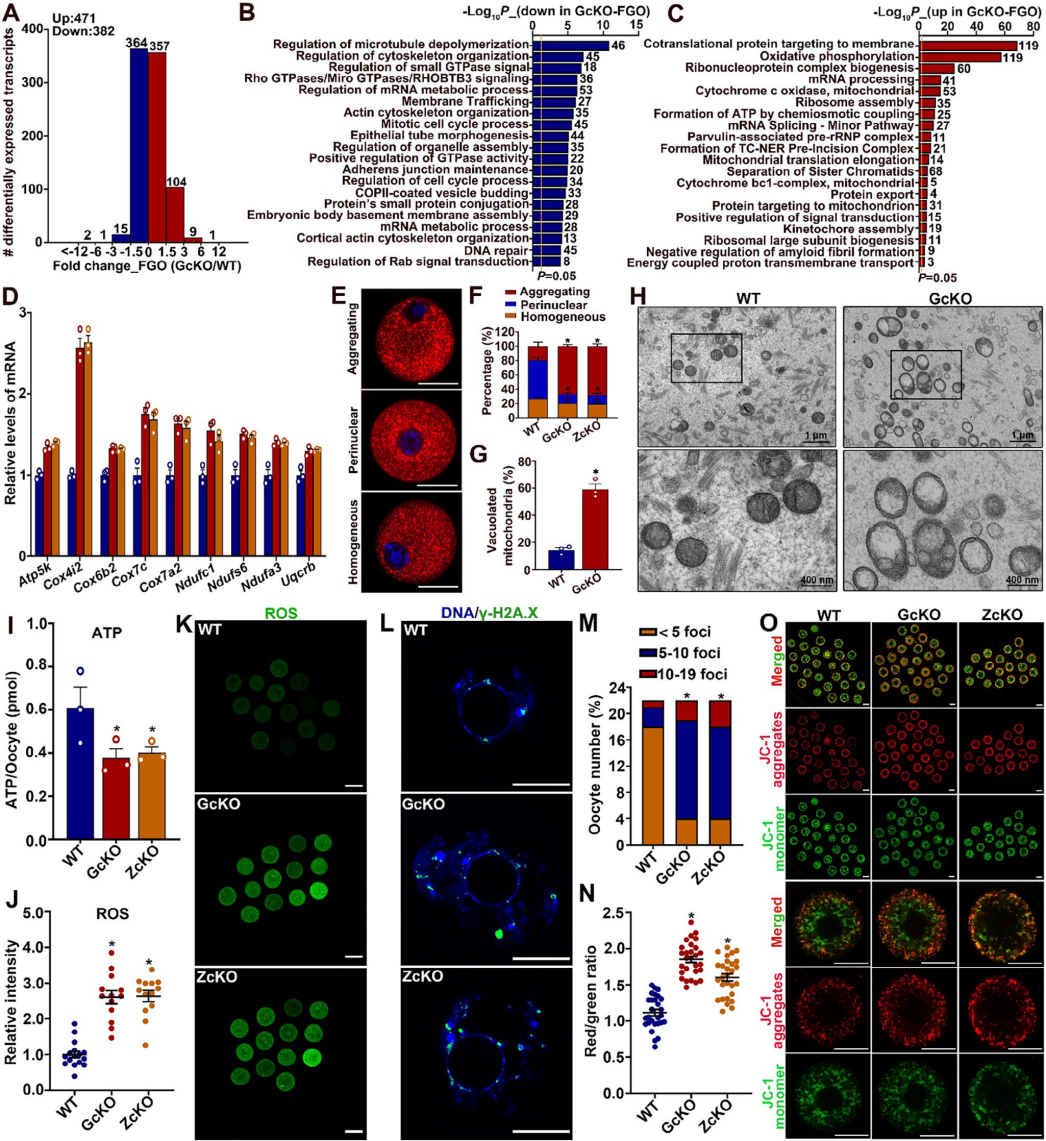
Impaired oocyte transcriptomic integrity and mitochondrial function in *Rspo2* oocyte‐specific knockout mice. A) Distribution of DEGs across varying magnitudes of expression change between WT and *Rspo2*‐GcKO FGOs as detected by RNA‐Seq. The number of genes in each category is indicated above each bar. B, C) Bar graphs showing the enriched pathways and biological processes associated with downregulated (B) and upregulated (C) genes in FGOs from *Rspo2*‐GcKO mice. D) qRT‐PCR validation of the upregulated expression of genes involved in mitochondrial function in FGOs from *Rspo2*‐cKO mice. Data are presented as Mean ± SEM (*N* = 3). ^*^ *p*<0.05, *Rspo2*‐cKO versus WT. E, F) Analysis of mitochondrial distribution in GV‐stage FGOs from *Rspo2*‐cKO mice by Mito Tracker labeling and live imaging: (E) Representative micrographs showing three distribution patterns — “aggregating”, “perinuclear”, and “homogeneous”. Scale bar = 50 µm; (F) Quantification of oocytes displaying each patterns. Experiments were independently repeated three times (15 oocytes/genotype/experiment). Data are shown as Mean ± SEM. ^*^ *p*<0.05, *Rspo2*‐cKO versus WT by χ^2^ test. G, H) Ultrastructural analysis of mitochondrial morphology in GV‐stage FGOs from *Rspo2*‐GcKO mice by transmission electron microscopy (TEM): (G) Quantification of the ratio of oocytes with vacuolated mitochondria. Eight semithin sections per genotype were analyzed. Data are shown as Mean ± SEM. ^*^ *p*<0.05, *Rspo2*‐cKO versus WT; (H) Representative TEM micrographs of mitochondrial morphology, with amplified views of the boxed area shown in the bottom panels. I) Comparison of ATP levels between GV‐stage FGOs from *Rspo2*‐cKO and WT mice. The experiment was independently repeated three times, using 50 oocytes per genotype per replicate. Data are presented as Mean ± SEM. ^*^ *p*<0.05, *Rspo2*‐cKO versus WT. J, K) Evaluation of reactive oxygen species (ROS) levels in GV‐stage FGOs from *Rspo2*‐cKO and WT mice: (J) Quantification of ROS levels in *Rspo2*‐cKO oocytes (a total of 13 oocytes were analyzed). Data are presented as Mean ± SEM. ^*^ *p*<0.05, *Rspo2*‐cKO versus WT; (K) Representative micrographs showing ROS staining (green) in oocytes. Scale bars = 100 µm. L, M) Analysis of DNA double‐strand breaks in oocytes by IF staining of γ‐H2A.X: (L) Representative micrographs showing γ‐H2A.X staining (green) with DNA counterstained with DAPI (blue); (M) Quantification of γ‐H2A.X‐positive oocytes, with 30 oocytes analyzed per genotype. Data are presented as Mean ± SEM. ^*^ *p*<0.05, *Rspo2*‐cKO versus WT by χ^2^ analysis. N, O) Evaluation of mitochondrial membrane potential levels in GV‐stage FGOs from *Rspo2*‐cKO and WT mice by JC1 staining: (N) Quantification of mitochondrial membrane potential levels in *Rspo2*‐cKO oocytes. A total of 28 oocytes were evaluated. Data are presented as Mean ± SEM. ^*^ *p*<0.05, *Rspo2*‐cKO versus WT; (O) Representative micrographs showing oocytes labeled with the JC1 probe, with the magnified view of the oocyte shown in the bottom panels. Scale bars = 100 µm.

We then performed transcriptomic analysis on FGOs isolated from WT and *Rspo2*‐GcKO mice to unravel potential molecular changes underlying the decline in oocyte quality in *Rspo2*‐cKO mice. A total of 853 genes were found to be differentially expressed in the *Rspo2*‐GcKO FGOs, with 382 genes downregulated and 471 genes upregulated (**Figure** **8A**; Table S6, Supporting Information). Gene enrichment analysis revealed that the most significant biological processes associated with the upregulated genes were related to “mitochondrial function,” while “actin/microtubule cytoskeleton/organelle assembly” was primarily associated with the downregulated genes (Figure 8B,C). Interestingly, both the up‐ and downregulated genes were also enriched in “mRNA metabolism” and “mitosis/cell cycle regulation” processes (Figure 8B,C).

Notably, nine genes encoding key enzymes involved in mitochondrial oxidative phosphorylation and ATP production were significantly upregulated in the *Rspo2*‐GcKO FGOs, as confirmed by qRT‐PCR (Figure 8D). Although these genes are crucial for mitochondrial function, their upregulation may have detrimental effects on mitochondrial health. To investigate this further, we examined potential changes in mitochondrial morphology and function in *Rspo2*‐GcKO FGOs. MitoTracker labeling and live‐cell imaging revealed alterations in mitochondrial distribution patterns, with more oocytes showing a uniform aggregation of mitochondria into clusters within the cytoplasm at both the GV and MII stages (Figure 8E,F; Figure S7B, Supporting Information). Transmission electron microscopy analysis further confirmed these changes, revealing significant ultrastructural alterations in the mitochondria of *Rspo2*‐GcKO GV‐stage oocytes (Figure 8G,H; Figure S7A, Supporting Information). Approximately 60% of the mitochondria in *Rspo2*‐GcKO oocytes exhibited large vacuoles and swelling, compared to less than 20% in WT oocytes (Figure 8G).

In line with these morphological changes, mitochondrial function was also impaired, as indicated by decreased ATP levels and elevated reactive oxygen species (ROS) content in the *Rspo2*‐cKO oocytes (Figure 8I–K; Figure S7C,D, Supporting Information). Correspondingly, DSBs, measured by IF staining of γH2AX, were significantly increased in *Rspo2*‐cKO oocytes (Figure 8L,M). Interestingly, despite these mitochondrial dysfunctions, mitochondrial membrane potential was unexpectedly elevated in *Rspo2*‐cKO oocytes (Figure 8N,O; Figure S7E,F, Supporting Information). Despite these mitochondrial and transcriptomic alterations, no overt meiotic abnormalities were observed in the matured *Rspo2*‐cKO oocytes. Meiotic progression, as assessed by spindle morphology, chromosome alignment, and F‐actin distribution, appeared normal (Figure S7G,H, Supporting Information). Nevertheless, this disturbance in mitochondrial homeostasis likely contributes to the decline in oocyte quality and subsequent female fertility in *Rspo2*‐cKO mice.

### 2.7 RSPO2 Promotes *Amh* Transcription in Granulosa Cells by Binding to LGRs and Activating CTNNB1

**Figure 9:**
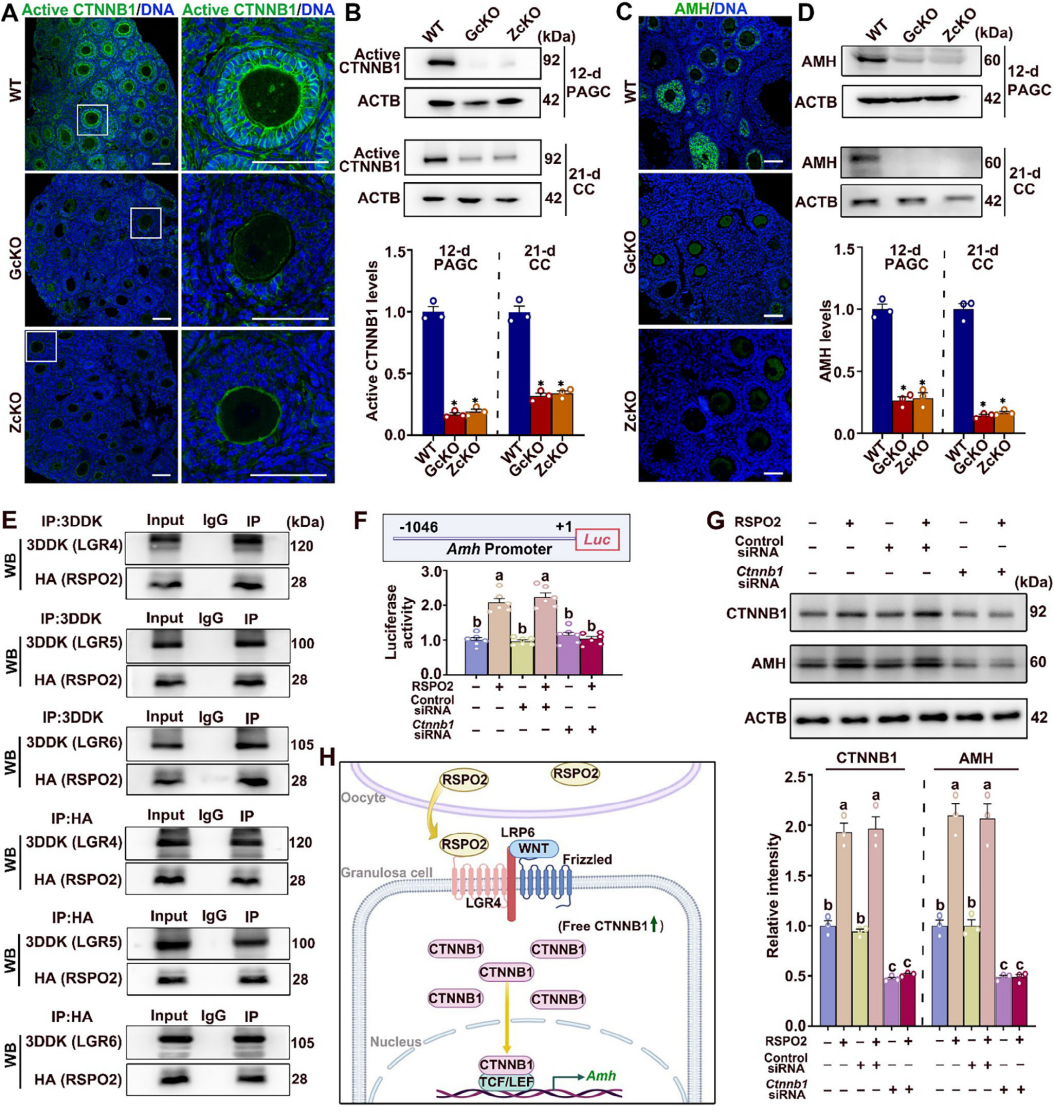
RSPO2 enhances *Amh* transcription in granulosa cells through LGR binding and CTNNB1 activation. A, B) RSPO2 deficiency reduces active CTNNB1 expression in granulosa cells: (A) Representative IF images showing active CTNNB1 (green) in ovaries from WT, *Rspo2*‐GcKO (GcKO), and *Rspo2*‐ZcKO (ZcKO) mice. Enlarged views of boxed regions are shown on the right. Nuclei are counterstained with DAPI (blue). Scale bars = 100 µm; (B) Western blot (WB) analysis of active CTNNB1 in PAGCs (from 12‐day‐old secondary follicles) and CCs (from 21‐day‐old large antral follicles). Representative blots are shown above, with quantification below. Data are presented as Mean ± SEM (*N* = 3). ^*^ *p*<0.05, *Rspo2*‐cKO versus WT. C, D) RSPO2 deficiency reduces AMH expression in granulosa cells: (C) Representative IF images showing AMH (green) in ovaries from WT, *Rspo2*‐GcKO (GcKO), and *Rspo2*‐ZcKO (ZcKO) mice. Nuclei are counterstained with DAPI (blue). Scale bars = 100 µm; (D) WB analysis of AMH in PAGCs and CCs. Representative blots are shown above, with quantification below. Data are presented as Mean ± SEM (*N* = 3). ^*^ *p*<0.05, *Rspo2*‐cKO versus WT. E) Co‐immunoprecipitation (Co‐IP) and WB analysis demonstrating the interaction between RSPO2 and LGR receptors: PAGCs were co‐transfected with HA‐tagged *Rspo2* and 3DDK‐tagged *Lgr4*, *Lgr5*, or *Lgr6*. Co‐IP was performed using an anti‐DDK antibody, followed by WB analysis to detect HA‐tagged RSPO2 protein, and vice versa. Representative blots confirm the interaction between RSPO2 and each LGR receptor. F) Luciferase reporter assay to evaluate *Amh* transcription regulation by RSPO2: The top panel shows the experimental design, where a 1047 bp DNA fragment (positions +1 to −1046) of the *Amh* promoter was cloned into a luciferase reporter vector and transfected into cultured monolayer PAGCs. The bottom panel displays luciferase activity results following RSPO2 treatment in cells transfected with either control siRNAs or *Ctnnb1*‐specific siRNAs. Data are presented as Mean ± SEM (*N* = 3). Groups marked with different letters are significantly different (^*^ *p*<0.05). G) Effect of *Ctnnb1* knockdown on RSPO2‐induced AMH expression in cultured PAGCs: WB analysis of AMH protein levels in PAGCs treated with or without RSPO2 following transfection with control or *Ctnnb1*‐specific siRNAs. The upper panel shows representative WB images; the lower panel quantifies AMH expression. Data are presented as Mean ± SEM (*N* = 3). Groups marked with different letters are significantly different (^*^ *p*<0.05). H) Schematic illustration of the proposed mechanisms: Oocyte‐secreted RSPO2 binds to LGR4 on granulosa cells and activates downstream CTNNB1 signaling, thereby regulating the transcription of target gene, such as *Amh*.

RSPO2 is known to function by binding to LGR receptors and potentiating the activation of CTNNB1 in various cell types. We therefore tested whether RSPO2 also act through the CTNNB1‐dependent pathway in ovarian granulosa cells. We performed IF staining and Western blot (WB) analyses of active CTNNB1 on ovarian sections and isolated granulosa cells, respectively. The results indeed showed that the levels of active CTNNB1 were significantly reduced in both the PAGCs of 12‐day‐old secondary follicles and the cumulus cells of 21‐day‐old large antral follicles from *Rspo2*‐GcKO and *Rspo2*‐ZcKO mouse ovaries (Figure **9**A,B).

Preceding experiments have demonstrated that RSPO2 treatment induces the expression of *Amh* mRNA in cultured granulosa cells, while *Rspo2*‐cKO in oocytes leads to a marked reduction in *Amh* mRNA levels in surrounding granulosa cells. Here, we confirmed the changes in *Amh* expression at the protein level by performing IF staining on ovarian sections and WB analysis on isolated granulosa cells. The results clearly showed that AMH protein was significantly reduced to nearly undetectable levels in both PAGCs and cumulus cells of *Rspo2*‐GcKO and *Rspo2*‐ZcKO mice (Figure 9C,D). Therefore, *Amh* is most likely a bona fide downstream target of RSPO2 in granulosa cells, with RSPO2 promoting its expression. Given the crucial role of AMH in regulating ovarian follicle development and female fertility, we used *Amh* as a representative downstream target to further investigate whether RSPO2 functions via the CTNNB1‐dependent pathway to regulate gene expression in granulosa cells.

We first tested whether RSPO2 binds to LGR receptors in granulosa cells. Co‐immunoprecipitation (Co‐IP) followed by WB analysis revealed that when the cDNA of *Rspo2* was co‐transfected with that of *Lgr4*, *Lgr5*, or *Lgr6* into cultured PAGCs, the ectopically expressed RSPO2 interacted with the exogenously expressed LGR proteins (Figure 9E). Next, we conducted a luciferase reporter assay to test whether RSPO2 regulates *Amh* transcription. A 1047 bp DNA fragment spanning positions +1 to ‐1046 of the *Amh* promoter was cloned into a luciferase reporter vector, and the resulting plasmid was transfected into cultured monolayer PAGCs. The results showed that RSPO2 stimulated *Amh* transcription in cultured PAGCs (Figure 9F). Furthermore, when CTNNB1 was specifically knocked down using siRNAs, both the basal and RSPO2‐induced levels of AMH in PAGCs were significantly reduced (Figure 9F,G). Collectively, these data support the model that oocyte‐secreted RSPO2 binds to its cognate LGR receptors, specifically LGR4, on the granulosa cell membrane, which activates the WNT‐CTNNB1 pathway and subsequently regulates the expression of its target genes, such as *Amh*, in granulosa cells (Figure 9H).

### 2.8 Crosstalk between RSPO2‐CTNNB1 and GDF9:BMP15‐SMAD2 Signaling Pathways in Granulosa Cells

The preceding experiments have demonstrated that oocyte‐derived RSPO2 coordinates with GDF9:BMP15 heterodimers to regulate granulosa cell gene expression. RSPO2 exerts its function, at least in part, by potentiating the activation of CTNNB1 signaling. On the other hand, GDF9:BMP15 heterodimers have been shown to activate the SMAD2/3‐dependent signaling pathways.^[^ ^8a,b^ ^]^ We hypothesized that the coordination between RSPO2 and GDF9:BMP15 heterodimers in regulating granulosa cell development occurs at an immediate downstream step after receptor binding—specifically, through the activation of SMAD2 and the prevention of CTNNB1 degradation.

**Figure 10:**
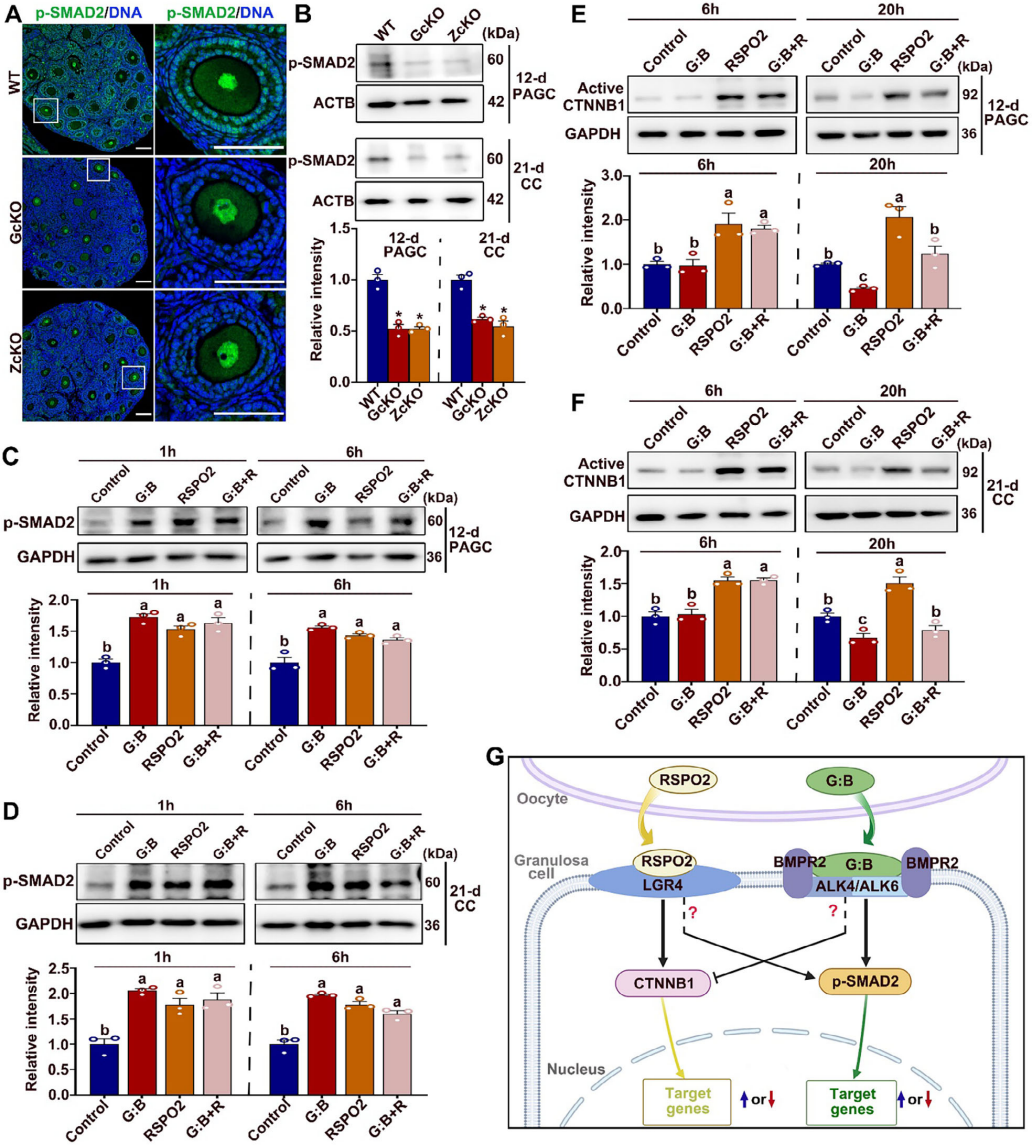
Crosstalk between RSPO2‐CTNNB1 and GDF9:BMP15‐SMAD2 signaling pathways in granulosa Cells. A,B) RSPO2 deficiency reduces active p‐SMAD2 expression in granulosa cells: (A) Representative IF images showing p‐SMAD2 (green) in ovaries from WT, *Rspo2*‐GcKO (GcKO), and *Rspo2*‐ZcKO (ZcKO) mice. Enlarged views of boxed regions are shown on the right. Nuclei are counterstained with DAPI (blue). Scale bars = 100 µm; (B) Western blot (WB) analysis of p‐SMAD2 in PAGCs (from 12‐day‐old secondary follicles) and CCs (from 21‐day‐old large antral follicles). Representative blots are shown above, with quantification below. Data are presented as Mean ± SEM (*N* = 3). ^*^ *p*<0.05, *Rspo2*‐cKO versus WT. C, D) WB analysis of p‐SMAD2 expression in cultured PAGCs (C) and CCs (D) treated with GDF9:BMP15 heterodimers (G:B), RSPO2, or both (G:B+R) for 1 or 6 h. Representative WB images are shown in the upper panels; quantification of p‐SMAD2 levels is presented in the lower panels. Data are expressed as Mean ± SEM (N = 3). Groups marked with different letters differ significantly (^*^ *p*<0.05). E, F) WB analysis of active CTNNB1 expression in cultured PAGCs (E) and CCs (F) treated with GDF9:BMP15 heterodimers (G:B), RSPO2, or both (G:B+R) for 6 or 20 h. Representative WB images are shown in the upper panels; quantification of active CTNNB1 levels is shown in the lower panels. Data are expressed as Mean ± SEM (N = 3). Groups marked with different letters differ significantly (^*^ *p*<0.05). G) Schematic representation of the crosstalk between the RSPO2‐CTNNB1 and GDF9:BMP15 (G:B)‐SMAD2 signaling pathways in granulosa cells. The “?” symbol denotes steps that remain to be elucidated.

To test this hypothesis, we first assessed whether SMAD2 activation, indicated by its phosphorylation, was altered in granulosa cells from *Rspo2* oocyte‐specific knockout mouse ovaries. IF staining revealed that the active, phosphorylated form of SMAD2 (p‐SMAD2) was nearly undetectable in the granulosa cells of both *Rspo2*‐GcKO and *Rspo2*‐ZcKO mouse ovaries compared to WT controls (**Figure** **10**A). WB analysis further confirmed a marked reduction in p‐SMAD2 expression in both PAGCs and cumulus cells isolated from *Rspo2*‐GcKO and *Rspo2*‐ZcKO mouse ovarian follicles (Figure 10B). These results suggest that oocyte‐secreted RSPO2 is required for proper activation of the SMAD2 signaling pathway in granulosa cells in vivo.

We next examined whether RSPO2 could directly activate SMAD2 in cultured granulosa cells. RSPO2 supplementation indeed promoted SMAD2 activation in cultured PAGCs and cumulus cells, reaching levels comparable to those induced by GDF9:BMP15 heterodimers (Figure 10C,D). This effect was observed at both 1 and 6 h after treatment. Notably, no additive effect on SMAD2 activation was observed when RSPO2 and GDF9:BMP15 heterodimers were combined in the culture medium (Figure 10C,D).

Finally, we investigated whether GDF9:BMP15 heterodimers could modulate RSPO2‐CTNNB1 signaling in cultured granulosa cells. PAGCs and cumulus cells were cultured with GDF9:BMP15 heterodimers alone or in combination with RSPO2 for short (6 h) and prolonged (20 h) durations. Active CTNNB1 levels were assessed by WB analysis. A separate group of granulosa cells treated with RSPO2 alone served as a positive control. As shown in Figure 10E, F, RSPO2 treatment significantly increased active CTNNB1 levels in cultured PAGCs and cumulus cells at both 6 and 20 h. However, 6‐h treatment with GDF9:BMP15 heterodimers had no effect on either basal or RSPO2‐induced levels of active CTNNB1. Surprisingly, 20‐h treatment with GDF9:BMP15 heterodimers resulted in a marked reduction of both basal and RSPO2‐induced active CTNNB1 levels in PAGCs and cumulus cells (Figure 10E,F).

Taken together, these findings indicate a significant interaction between the RSPO2‐CTNNB1 and GDF9:BMP15‐SMAD2 signaling pathways in granulosa cells. While RSPO2 promotes SMAD2 activation, GDF9:BMP15 heterodimers suppress the levels of active CTNNB1, highlighting a complex interplay between these signaling cascades in regulating granulosa cell function (Figure 10G).

## 3 Discussion

Granulosa cell‐specific gene expression is essential for granulosa cell development and the progression of oogenesis and folliculogenesis. This study identifies RSPO2 as a key OSF that, in coordination with GDF9:BMP15 heterodimers, fine‐tunes granulosa cell transcriptomes through gene‐specific synergistic and antagonistic interactions. Oocyte‐specific deletion of *Rspo2* in mice disrupted granulosa cell transcriptomic integrity and TZPs, impaired granulosa cell proliferation and survival, and compromised oocyte quality, fertility, and reproductive lifespan, highlighting the critical role of coordinated OSF signaling and oocyte‐granulosa cell crosstalk in follicular development.

Preantral follicle growth largely depends on local intra‐follicular cues rather than pituitary gonadotropins, with OSFs serving as key regulators.^[^ ^1d^ ^]^ While OSFs are known to promote granulosa cell proliferation and expression of essential genes like *Amh* and *Kitl*,^[^ ^1^, ^5^, ^15^ ^]^ their transcriptome‐wide impact on PAGCs remained unclear. Through comparative transcriptomic analyses of intact GOCs, OOX‐PAGCs, and OOX‐PAGCs co‐cultured with oocytes, we demonstrate that oocytes maintain the molecular architecture of PAGCs. OSFs primarily promote the expression of genes associated with cell proliferation (e.g., *Ccnd2*, a key driver of granulosa cell proliferation^[^ ^16^ ^]^) and metabolism (e.g., cholesterol and small molecule biosynthesis), while suppressing anti‐proliferative pathways (e.g., *Cdkn1c*, an inhibitor of CCND2^[^ ^17^ ^]^). These findings provide a comprehensive molecular framework for how oocytes drive granulosa cell development and preantral follicle growth, supporting earlier observations that oocytes promote granulosa cell proliferation and orchestrate the rate of follicular development.^[^ ^18^ ^]^

Granulosa cells, originating from the ovarian surface epithelium,^[^ ^19^ ^]^ maintain their cellular identity through expression of characteristic genes critical for oocyte and follicular development.^[^ ^20^ ^]^ These genes, including *Amh*, *Dhh/Ihh*, *Fst*, *Inhba/Inhbb*, *Kitl*, and *Nppc*, are indispensable for folliculogenesis, with their disruption leading to fertility defects.^[^ ^21^ ^]^ Our study demonstrates that oocyte removal significantly downregulates these transcripts in PAGCs, whereas co‐culture with oocytes restores their expression, highlighting the crucial role of OSFs in maintaining granulosa cell identity. Moreover, oocytes suppress expression of genes associated with extracellular matrix remodeling, cell migration, EMT, and inflammation—processes often associated with aging and cellular identity loss. These effects mirror transcriptomic changes observed in granulosa cells from aged ovaries across various species,^[^ ^22^ ^]^ suggesting OSFs play a protective role against granulosa cell aging and identity erosion. Supporting this notion, *Cldn5*, a Sertoli cell marker,^[^ ^23^ ^]^ was upregulated in OOX‐PAGCs, consistent with our prior report of granulosa‐to‐Sertoli‐like transdifferentiation following oocyte‐specific *Mtor* deletion.^[^ ^24^ ^]^ Collectively, these findings establish a dual mechanism by which oocytes maintain granulosa cell identity: (1) sustaining expression of developmental genes essential for folliculogenesis, and (2) suppressing transcriptional programs associated with cellular dysfunction, aging, and transdifferentiation.

We identified RSPO2 as a key OSF that, together with GDF9:BMP15 heterodimers, fine‐tunes the transcriptome of PAGCs, contributing significantly to the establishment of PAGC molecular architecture. While both factors promote granulosa cell development and identity, RSPO2 preferentially enhances anabolic gene expression, and GDF9:BMP15 primarily influences genes involved in mitosis and cell proliferation. This division of labor likely supports the distinct metabolic and proliferative demands of growing follicles.^[^ ^8^, ^25^ ^]^ GDF9:BMP15 heterodimers (cumulin) also promote anabolic gene expression in cumulus cells at later‐stage follicles,^[^ ^26^ ^]^ though RSPO2's role in these stages remains unclear. Importantly, RSPO2 and GDF9:BMP15 regulate some genes synergistically (e.g., *Amh*, *Ccnd2*, and *Fst*) and others antagonistically (e.g., *Amhr2*, *Kitl*, *Nppc*, and *Slc38a3*), revealing a target‐specific coordination mechanism. This duality helps reconcile earlier observations that oocytes promote *Kitl* expression, yet GDF9 suppresses it.^[^ ^15^, ^27^ ^]^ Unlike prior reports of OSF cooperation (e.g., GDF9 and BMP15, BMP15 and FGF8),^[^ ^8a,b^ ^]^ this gene‐dependent synergy and antagonism provide new insight into how oocytes precisely calibrate granulosa cell gene expression to support the coordinated development of both.

Consistent with the crucial role of RSPO2 and GDF9:BMP15 heterodimers in regulating granulosa cell gene expression, oocyte‐specific deletion of *Rspo2* disrupted granulosa cell transcriptomes, impaired folliculogenesis, and led to subfertility and premature reproductive failure. Compared to previous *ex vivo* and transplantation‐based studies,^[^ ^12^, ^14^ ^]^ our oocyte‐specific cKO model more faithfully recapitulates the physiological role of oocyte‐secreted RSPO2. RSPO2 is required not only for the primary‐to‐secondary follicle transition but also for maintaining the ovarian reserve, supporting later‐stage follicle development, and ensuring oocyte competence. Similar phenotypes observed in both *Rspo2‐*GcKO and *Rspo2*‐ZcKO models suggest that RSPO2 acts postnatally in growing follicles, consistent with its expression pattern.

Transcriptomic analyses of *Rspo2*‐GcKO granulosa cells revealed gene regulatory alterations associated with impaired granulosa cell proliferation, follicle attrition, and compromised oocyte quality. In PAGCs, *Ccnd2* downregulation and *Cdkn1* upregulation likely underlie reduced cell proliferation, consistent with their established roles in follicle growth and fertility.^[^ ^16^, ^17^ ^]^ Reduced expression of *Amh* and *Kitl* may further contribute to premature follicle depletion and shortened reproductive lifespan, as AMH restricts primordial follicle recruitment,^[^ ^21b^ ^]^ and KIT signaling in oocytes is critical for follicle survival.^[^ ^21^, ^28^ ^]^ Notably, RSPO2‐regulated genes in cumulus cells differed markedly from those in PAGCs, displaying limited overlap and often opposing patterns. These observations suggest that RSPO2 exerts distinct, stage‐ and cell‐type‐specific effects, potentially modulated by other OSFs and granulosa cell differentiation status. The upregulation of diverse transcription factors in cumulus cells further points to a potential role for RSPO2 in modulating cumulus cell differentiation and preovulatory responsiveness.

Transcriptomic and functional alterations in cumulus cells and oocytes from *Rspo2*‐GcKO mice likely explain the observed loss of oocyte competence. Of 30 amino acid metabolism genes downregulated in cumulus cells, 27 are normally enriched in cumulus cells over oocytes, underscoring RSPO2's role in facilitating metabolic cooperation between the two compartments. This supports prior findings that oocytes promote *Slc38a3* expression to mediate amino acid transport in cumulus cells.^[^ ^25^ ^]^ In *Rspo2*‐GcKO cumulus cells, downregulation of key developmental regulators, coupled with increased apoptotic and anti‐proliferative transcripts, likely impairs their support for oocytes. Correspondingly, oocytes displayed reduced developmental competence and mitochondrial abnormalities in distribution, ultrastructure, and function. Although mitochondrial respiratory genes were upregulated, likely reflecting a compensatory response, these molecular and organellar defects underscore the essential role of RSPO2‐mediated somatic support in sustaining oocyte quality.

We demonstrate that RSPO2 activates both CTNNB1 and SMAD2 signaling in granulosa cells, whereas GDF9:BMP15 heterodimers activate only SMAD2 and antagonize RSPO2‐induced CTNNB1 activation. The mechanisms underlying this coordinated crosstalk between the RSPO2–CTNNB1 and GDF9:BMP15–SMAD2 pathways in granulosa cells remains unclear. They may be explained by the recently proposed “R‐spondin code” hypothesis,^[^ ^29^ ^]^ which proposes that distinct motifs within the TSP1 domains of RSPOs enable selective modulation of downstream pathways. RSPO2 may differentially regulate WNT and other pathways via TSP1‐mediated internalization of BMPR1A or FGFR4 through interactions with ZNRF3/RNF43.^[^ ^30^ ^]^ Although untested in granulosa cells, this model provides a compelling framework for RSPO2 function in coordinating GDF9:BMP15 and potentially other OSF signaling during folliculogenesis.^[^ ^31^ ^]^

## 4 Conclusion

This study reveals a sophisticated mechanism of oocyte–granulosa cell communication essential for follicular development. By uncovering the coordinated actions of RSPO2 and GDF9:BMP15 heterodimers, we define key signaling and transcriptional programs that regulate granulosa cell function and oocyte competence. Beyond expanding our understanding of oocyte‐derived signaling, this work lays a foundation for future therapeutic strategies targeting OSF signaling to treat ovarian dysfunction and preserve fertility.

## 5 Experimental Section

### Mice and Fertility Test

Mice carrying the conditional allele of *Rspo2* (C57BL/6JCya‐*Rspo2* ^em1flox^/Cya, hereafter referred to as *Rspo2* ^fl/fl^) were generated by the Cyagen Biosciences (Suzhou) Incorporation (Taikang, Jiangsu, China). The Tg(*Gdf9*‐icre)5092Coo and Tg(*Zp3*‐cre)93Knw (hereafter referred to as *Gdf9*‐Cre/+ and *Zp3*‐Cre/+, respectively) mice were obtained from The Jackson Laboratory (Bar Harbor, ME). To generate the oocyte‐specific knockout (KO) of *Rspo2* female mice for experiments, *Rspo2* ^fl/fl^ female mice were first crossed with *Gdf9*‐Cre/+ and *Zp3*‐Cre/+ males, respectively, to produce *Rspo2* ^fl/+^;*Gdf9*‐Cre/+ and *Rspo2* ^fl/+^;*Zp3*‐ Cre/+ progenies. The males of these progenies were then mated with *Rspo2* ^fl/fl^ females to produce *Rspo2* ^fl/fl^;*Gdf9*‐Cre/+ and *Rspo2* ^fl/fl^;*Zp3*‐Cre/+ mice, respectively. The resultant *Rspo2* ^fl/fl^;*Gdf9*‐Cre/+ and *Rspo2* ^fl/fl^;*Zp3*‐Cre/+ male mice were routinely crossed with *Rspo2* ^fl/fl^ females to generate *Rspo2* ^fl/fl^ (wild‐type, WT), *Rspo2* ^fl/fl^;*Gdf9*‐Cre/+ (*Rspo2*‐GcKO), and *Rspo2* ^fl/fl^;*Zp3*‐Cre/+ (*Rspo2*‐ZcKO) conditional knockout (cKO) females for use in this study. Mouse genotypes were determined by PCR using primers listed in Table S1 (Supporting Information).

Fertility tests were executed by mating 8‐week‐old cKO female mice and their WT female littermates with normal adult C57BL/6J x DBA2 F1 (B6D2F1) males for a period of 10 months. The number of pups born per litter was recorded at birth, and the accumulated number of pups, as well as the average litter sizes during the 2–8 months and 8–12 months period of breeding, were calculated.

Mice used in experiments other than these genetic studies belong to the inbred Kunming strain, which were purchased from Qingdao Darenfucheng Animal Technology Co., Ltd (Qingdao, Shandong, China). All mice were housed under the standard conditions at the animal facility of Shandong university. All procedures and protocols involving mice were approved by the Institutional Animal Care and Use Committee of Shandong university (approval number: SYDWILL‐2023‐024).

### Reagents and Chemicals

Unless otherwise specified, all reagents and chemicals used in this study were purchased from Sigma‐Aldrich (St. Louis, Missouri, USA)

### Isolation and Culture of Granulosa Cells and Oocytes

Oocytes at the primordial follicle and primary follicle stages were isolated from ovaries of neonatal mice at the age of day 3 and 6, respectively, as described previously.^[^ ^32^ ^]^ Preantral granulosa cell‐oocyte complexes (GOCs) were isolated from the secondary follicles of 12‐day‐old female mice following previously established protocols.^[^ ^33^ ^]^ Briefly, ovaries were enzymatically digested with 4 mg/ml collagenase in bicarbonate‐buffered MEM‐alpha (Cat# C11095500BT, Thermo Fisher Scientific Inc., Waltham, Massachusetts, USA) with Earles’ salts, supplemented with 75 µg mL^−1^ penicillin G, 50 µg mL^−1^ streptomycin sulfate, 0.23 mM pyruvate, and 3 mg/ml bovine serum albumin at 37 °C, and GOCs with 2–3 layers of granulosa cells were carefully dissociated individually from the ovarian tissue. Growing oocytes (GOs), approximately 56 um in diameter, were then isolated by digesting these GOCs with 4 mg/ml collagenase in PBS (pH 7.2) at 37 °C, followed by gentle pipetting with a 1 ml pipette to remove the surrounding granulosa cells.^[^ ^33^ ^]^ Cumulus‐oocyte complexes (COCs) were isolated from 22‐day‐old females primed with eCG for 46 h by puncturing large antral follicles on the surface of the ovaries. Fully‐grown oocytes (FGOs), approximately 76 µm in diameter, were obtained by stripping off the cumulus cells from COCs using a fine‐bore glass pipette with an inner diameter slightly narrower than that of the oocyte.^[^ ^33^ ^]^ To investigate the effect of oocytes on preantral granulosa cells (PAGCs) and cumulus, oocytes were microsurgically removed from GOCs and COCs through OOX. This procedure was performed using micromanipulators under an inverted microscope, following previously described protocols.^[^ ^34^ ^]^ Granulosa cells and oocytes were cultured in the same bicarbonate‐buffered MEM‐alpha (Thermo Fisher Scientific) medium used for oocyte and granulosa cell isolation at 37 °C and 100% humidity in an Esco CelCulture CCL‐170T‐8‐IVF incubator (Esco Micro Pte. Ltd, Singapore, Singapore) infused with 5% O_2_, and 5% CO_2_.

### Oocyte In Vitro Fertilization and Embryo Culture

In vivo oocyte maturation was induced in 21‐day‐old female mice by intraperitoneal injection of 5 IU/mouse equine chorionic gonadotropin (eCG, cat# 110914564, SANSHENG, Ningbo, China), followed 48 h later by 5 IU/mouse human chorionic gonadotropin (hCG; Cat# 110911282, SANSHENG) for an additional 14 h. Matured oocytes were collected by carefully dissecting the ampulla region of the oviduct, allowing the release of expanded COCs into bicarbonate‐buffered MEM medium (Cat# 12000–022, Thermo Fisher Scientific) supplemented with Earle's salts, 75 µg mL^−1^ penicillin G, 50 µg mL^−1^ streptomycin sulfate, 0.23 mM pyruvate, and 3 mg/ml bovine serum albumin (BSA). In vitro fertilization (IVF) was performed by inseminating the ovulated COCs with sperm isolated from adult B6D2F1 males. Fertilization success was confirmed by the presence of pronuclei at 8 h and 2‐cell stage embryos at 24 h post‐IVF. Embryos were then cultured in KSOM medium, and development to the 4‐cell, morula, and blastocyst stages was assessed on days 3, 4, and 5 post‐IVF, respectively.

### RNA‐Seq Analysis

To investigate the influence of oocytes and OSFs on the transcriptome of PAGCs, 100 GOCs, 150 OOX‐PAGCs, and 100 OOX‐PAGCs co‐cultured with 400 GOs were cultured in 100 µl MEM‐alpha medium for 48 h. To further evaluate the effect of RSPO2 and GDF9:BMP15 heterodimers (G:B) on the PAGC transcriptome, 150 OOX‐PAGCs were cultured under the following conditions for 48 h: untreated control, treatment with 50 ng mL^−1^ RSPO2 (cat# 6946‐R, R&D Systems, Minneapolis, Minnesota, USA), treatment with 30 ng mL^−1^ G:B (a kind gift from Dr. Martin Matzuk at Baylor College of Medicine), and treatment with a combination of 50 ng mL^−1^ RSPO2 and 30 ng mL^−1^ G:B. The doses of RSPO2 and GDF9:BMP15 used in this study were selected based on the preliminary tests, which identified them as optimally effective. Following the culture period, PAGCs from each group were collected in 350 µl RLT lysis buffer for subsequent RNA extraction and analysis. To analyze transcriptomic changes in oocytes and granulosa cells from *Rspo2*‐GcKO female mice, three sets of FGOs, cumulus cells, and PAGCs were collected. Each set consisted of 80 GV‐stage FGOs, cumulus cells from 80 COCs, and PAGCs from 80 GOCs, respectively. All collected samples were preserved in 350 µl RLT lysis buffer for RNA extraction and further analysis.

Total RNA was extracted from the collected samples using the RNeasy Micro Kit (Cat# 74 004, Qiagen, Hilden, North Rhine‐Westphalia, Germany) following the manufacturer's instructions. Poly(A) mRNA isolation, mRNA library construction, and subsequent sequencing and data processing were performed as described previously.^[^ ^21f^ ^]^ Differentially expressed genes (DEGs) were identified using the default parameters of Cuffdiff (v2.2.1). Only genes with an FPKM value of at least 5 in a minimum of three samples were included for further statistical analysis. Significantly altered genes were defined by the criteria of FDR *P* < 0.05 and |fold change| ≥ 1.5. The RNA‐Seq data have been deposited in the National Center for Biotechnology Information (NCBI) Sequence Read Archive (SRA) under the accession number BioProject PRJNA1181298. Gene enrichment analysis of DEGs was performed using Metascape (http://metascape.org).

### Genomic DNA Extraction and Genotyping PCR

Genomic DNA was extracted using the Blood/Cell/Tissue Genomic DNA Extraction Kit (Cat# DP304, Tiangen Biotech, Beijing, China) following the manufacturer's protocol with minor modifications. Samples were homogenized in lysis buffer (Buffer GA) containing proteinase K and incubated at 56 °C until digestion was complete. The lysates were mixed with absolute ethanol and transferred to adsorption columns. Contaminants were removed by sequential washing with GD (wash buffer 1) and PW (wash buffer 2), followed by centrifugation at 12 000 × g. Purified DNA was eluted in 50 µL preheated TE buffer. DNA concentration and purity were assessed using a NanoDrop spectrophotometer (A260/A280 ratio ≥ 1.8). Genotyping PCR was then carried out using the purified genomic DNA and primer pairs listed in Table S1 (Supporting Information).

### Quantitative RT‐PCR Analysis

Total RNA was extracted using the Fast Pure Cell/Tissue Total RNA Isolation Kit (Cat # RC101‐01, Vazyme, Nanjing, China) following the manufacturer's instructions. Reverse transcription was performed using the HiScript III RT Supermix for qRT‐PCR (Cat# R323‐01, Vazyme), and qRT‐PCR was conducted with the AceQ Universal SYBR Master Mix (Cat# Q511‐02, Vazyme) reagent. The sequences of the primer pairs used in the study are listed in Table S1 (Supporting Information). Relative fold changes in mRNA levels were calculated using the 2^−ΔΔCt^ method, with *Rpl19* serving as the internal control. In experiments comparing mRNA levels between GV‐ and MII‐stage FGOs, as well as during various stages of preimplantation embryo development, an equal number of oocytes and embryos were used in each sample. Additionally, exogenous mKate mRNA was added during RNA extraction to serve as an external control for normalization of fold changes.

### Ovarian Histology and Follicle Count

Ovaries from *Rspo2*‐cKO and WT littermate control female mice at various ages (21 days, 2, 4, 6, 8, 10, 12, and 14 months) were collected, fixed in Bouin's fixative (Cat# HT10132, Sigma‐Aldrich), and embedded in paraffin. Serial sections of 5 µm thickness were prepared and stained with periodic acid‐Schiff (PAS) reagent and Lillie–Mayer hematoxylin to visualize ovarian structures. Follicles at different developmental stages were counted in every third section throughout the entire ovary. Follicle classification and quantification were performed based on the morphological criteria described previously.^[^ ^35^ ^]^

### Immunofluorescence and Confocal Microscopy

IF staining was performed as described previously.^[^ ^36^ ^]^ For ovarian IF, freshly isolated ovaries from 3‐day‐old, 12‐day‐old or 21‐day‐old female mice were fixed in 4% paraformaldehyde (PFA, Cat# 441244, Sigma–Aldrich) prepared in PBS at 4 °C overnight, followed by embedding in paraffin and sectioning at 5 µm thickness. The sections were incubated with primary antibodies at 4 °C overnight, followed by incubation with Alexa Fluor 594‐ or 488‐conjugated secondary antibodies (Thermo Fisher Scientific) for 2 h at room temperature. DNA was counterstained with Hoechst 33342 (2 µg mL^−1^) (Cat#14533, Sigma–Aldrich) for 10 min. The primary antibodies used for ovarian IF staining included: KI‐67 (Cat# ab16667, 1:200, Abcam, Cambridge, Cambridgeshire, UK); ZP2 (Cat# sc‐32752, 1:500, Santa Cruz Biotechnology, Dallas, Texas, USA); γ‐H2A.X (Cat# BS4760, 1:1000, Bioworld Technology, Dublin, Ohio, USA); DDX4 (Cat# ab13840, 1:500, Abcam); FOXL2 (Cat# NB100‐1277, 1:300, Novus Biologicals, Littleton, Colorado, USA); RDX (Cat# F1012, 1:200, Selleck Chemicals, Houston, Texas, USA); p‐ERM (Cat# 3726, 1:200, Cell Signaling Technology, Danvers, Massachusetts, USA); Active‐CTNNB1 (Cat# 05–665, 1:500, Sigma‐Aldrich); AMH (Cat# 14461‐1‐AP, 1:200, Proteintech, Wuhan, Hubei, China); and Phospho‐SMAD2 pSer465/pSer467 (Cat# 40–0800, 1:200, Thermo Fisher Scientific).

To quantify the number of oocytes within the primordial follicle pool in the postnatal newborn ovaries, ovarian serial sections from 3‐day‐old *Rspo2*‐GcKO and WT littermate control female mice were prepared and double‐stained using IF with anti‐DDX4 and FOXL2 antibodies. Oocyte were counted in every fifth section, and the total number of oocytes per ovary was then estimated by multiplying the sum of oocytes by 5, as described previously.^[^ ^37^ ^]^

For oocyte IF, samples were fixed in 4% PFA in PBS at room temperature for 30 min, followed by blocking for 1 h in PBS containing 0.1% Triton X‐100 (Sigma) and 10% fetal bovine serum (FBS) (Cat# A5670701, Thermo Fisher Scientific). The samples were then incubated with primary antibodies at 4 °C overnight or Rhodamine Phalloidin (1:750, Cat# R415, Thermo Fisher Scientific, final concentration 7.5 nM) at room temperature for 30 min, protected from light. After three washes with PBS, the samples were incubated with Alexa Fluor 594‐ or 488‐conjugated secondary antibodies (Thermo Fisher Scientific) for 2 h at room temperature. DNA was counterstained with Hoechst 33342 (2 µg mL^−1^) for 10 min to visualize chromosomes. The primary antibodies used for oocyte IF included: γ‐H2A.X (Cat# BS4760, 1:1000, Bioworld Technology); Monoclonal anti‐β‐tubulin (Cat# T4026, 1:600, Sigma‐Aldrich).

To visualize TZPs, oocytes were fixed for 15 min at room temperature (RT) in freshly prepared 2% PFA in PBS, then washed with PBST (PBS with 0.1% Tween‐20). Oocytes were incubated for 1 hr at RT in Rhodamine Phalloidin (final concentration, 10 nM). Oocytes were washed in PBST overnightat 4 °C, then mounted on glass slides using antifade with DAPI (Cat# P36931, Thermo Fisher Scientific).

Both ovarian and oocyte specimens were examined and imaged using a Nikon Ti2‐E‐C2+ laser scanning confocal microscope (Nikon, Tokyo, Japan).

### Apoptosis Assay by TUNEL Staining

Cellular apoptosis in ovarian tissue sections was assessed using TUNEL staining. Ovarian sections were prepared following the same procedure as for IF analysis. TUNEL staining was performed using the TUNEL BrightRed Apoptosis Detection Kit (Cat# A113‐01, Vazyme, China), according to the manufacturer's instructions and as described previously.^[^ ^38^ ^]^ DNA was counterstained with Hoechst 33342 (2 µg mL^−1^) to visualize nuclei. Images were acquired using a confocal microscope.

### Transmission Electron Microscopy (TEM)

GV‐stage FGOs were fixed in 2.5% glutaraldehyde for 2 h at 4 °C, followed by three washes with phosphate‐buffered saline (PBS) containing 1% polyvinyl alcohol for 15 min each. To facilitate identification, the oocyte samples were briefly stained with eosin for 15 seconds and subsequently embedded in 1.5% agarose (Cat# BS081, biosharp, Hefei, Anhui, China). The agarose‐embedded samples were trimmed into 2 × 2 × 2 mm cubes and further fixed overnight in 2.5% glutaraldehyde. The processed samples were then submitted to the Imaging Core Facility on campus for transmission electron microscopy (TEM) processing and imaging following standard protocols.

### Live Cell Labeling and Imaging of Mitochondria, Reactive Oxygen Species (ROS), and Mitochondrial Membrane Potential

GV‐ and MII‐stage FGOs were cultured in bicarbonate‐buffered MEM‐alpha medium, the same medium used for PAGC and oocyte culture, containing 0.1% cell‐permeant MitoTracker Red CMXRos (Cat# C1049B, Beyotime, Shanghai, China), 0.1% DCFH‐DA from the ROS Assay Kit (Cat# S0033S, Beyotime), or JC‐1 working solution (Cat# C2006, Beyotime) for 30 min (MitoTracker and ROS labeling) or 20 min (JC‐1 staining) at 37 °C in a humidified atmosphere of 5% CO₂. Milrinone (Cat#HY‐14252/CS‐1367, MedChemExpress, Monmouth Junction, New Jersey, USA) was added into the GV‐stage oocyte culture medium at a final concentration of 5 µM to help maintain the meiotic arrest. After incubation, oocytes were washed three times with fresh culture medium for 5 min each to remove excess dye. Fluorescence imaging was conducted using a confocal microscope. Quantification of fluorescence intensity was conducted using ImageJ software (National Institutes of Health, USA), ensuring consistent image processing parameters across all samples.

### Measurement of ATP Content

Oocyte ATP content was measured using the Enhanced ATP Assay Kit (Cat# S0027, Beyotime, China) following the manufacturer's instructions. Briefly, 50 oocytes were lysed on ice in 100 µL of lysis buffer provided in the kit. The standard reaction solution was prepared according to the manufacturer's guidelines. To eliminate background ATP, 100 µL of ATP detection working solution was added to each well and incubated at room temperature for 5 min. Subsequently, 20 µL of the oocyte lysate was added to the well, mixed thoroughly, and chemiluminescence was measured using a luminometer at least 2 seconds after mixing. A 6‐point standard curve (0.004, 0.02, 0.1, 0.5, 2.5, and 5 µM ATP) was included in each assay, and ATP content was quantified using the formula derived from the linear regression of the standard curve.

### Luciferase Assay

The *Amh* promoter region (‐1046 to +1 bp) was cloned into the pLucmiRNA vector, in which the Renilla coding region, *Amh* promoter, and luciferase coding region were incorporated into the same vector through a modified construct. Recombinant plasmids were confirmed by Sanger sequencing. Mouse granulosa cells at 50–60% confluence were transfected with 1.0 µg of the plasmid along with 20 µM *Ctnnb1* siRNA or control siRNA using Lipo6000 transfection reagent (Cat # C0526, Beyotime, China). After transfection, granulosa cells were cultured for 24 h, followed by treatment with or without RSPO2 for an additional 24 h. Luciferase activity was measured using the Double‐Luciferase Reporter Assay Kit (Cat # FR201, TransGen Biotech, Beijing, China) according to the manufacturer's protocol.

### Co‐Immunoprecipitation (Co‐IP)

The *Rspo2* coding region was cloned into the pCMV6‐HA vector (Cat# 631604, Clontech, Mountain View, California, USA), while the *Lgr4*, *Lgr5*, and *Lgr6* coding regions were cloned into the pCMV6‐AC‐3DDK vector (Cat# PS100057, Origene, Rockville, Maryland, USA). Mouse granulosa cells at 50–60% confluence were transfected with 1.0 µg of each plasmid using Lipo6000 transfection reagent (Cat # C0526, Beyotime, China). Cells were harvested 48 h post‐transfection, and a total of 10⁷ granulosa cells were lysed using the lysis buffer supplemented with 1% protease inhibitor cocktail from the Pierce Crosslink Immunoprecipitation Kit (Cat # 26147, Thermo Fisher Scientific). Immunoprecipitation (IP) was performed using either anti‐Flag antibody (Cat # F1804, Sigma) or anti‐HA antibody (Cat # 901502, BioLegend, San Diego, California, USA), followed by Western blot with the indicated antibodies to detect protein interactions.

### Western Blot Analysis

Oocytes, granulosa cells, or cumulus cells were lysed in 2 × Laemmli sample buffer (Bio‐Rad) and heated at 108 °C for 5 min. Denatured proteins were separated on SDS‐PAGE gels and subsequently transferred onto polyvinylidene difluoride (PVDF) membranes for protein detection. The membranes were then blocked with 5% non‐fat milk in TBST for 1 h at room temperature, followed by overnight incubation with primary antibodies at 4 °C. The primary antibodies used included: Anti‐beta‐actin (ACTB) antibody (Cat# 66009‐1‐Ig, 1:1000, Proteintech), Anti‐GAPDH antibody (Cat# 60004‐1‐Ig, 1:1000, Proteintech), Anti‐CTNNB1 antibody (Cat# 610153, 1:1000, BD Transduction Laboratories, San Jose, California, USA), Anti‐active‐CTNNB1 antibody (Cat# 05–665, 1:1000, Sigma‐Aldrich), Anti‐phospho‐SMAD2 (pSer465/pSer467) antibody (Cat# 40–0800, 1:500, Thermo Fisher Scientific), and Anti‐AMH antibody (Cat# 14461‐1‐AP, 1:500, Proteintech, China). Following three washes with TBST, the membranes were incubated with HRP‐conjugated secondary antibodies (Cat# SA00001‐2/SA00001‐1, Proteintech) for 2 h at room temperature. After additional washes, signals were detected using the ECL Western Blotting Substrate (Cat# MA0186, Meilunbio, Dalian, China) and visualized using the Micro Q9 Light Imaging System. ACTB or GAPDH served as internal loading controls.

### Statistical Analysis

All statistical analyses were conducted using GraphPad Prism software (GraphPad Software, Inc., USA). Data are presented as Mean ± SEM from at least three independent experiments. Statistical significance was determined as follows: For comparisons between two groups, Student's t‐test was used; For comparisons among multiple groups, one‐way ANOVA followed by Tukey's Honestly Significant Difference (HSD) test was applied. A *P*‐value of < 0.05 was considered statistically significant.

### Ethics Approval Statement

All procedures and protocols involving mice were approved by the Institutional Animal Care and Use Committee of Shandong university.

## Conflict of Interest

The authors declare no conflict of interest.

## Author Contributions

Y.‐Q.S. and Y.W. conceived the study; Y.W., H.L., L.Y., J.B., S.W., and Z.S. performed the research; Y.W. and Y.‐Q.S. analyzed the data; and Y.W. and Y.‐Q.S. wrote the paper.

## Supporting information



Supporting Information

Supplemental Table 1

Supplemental Table 2

Supplemental Table 3

Supplemental Table 4

Supplemental Table 5

Supplemental Table 6

## Data Availability

The data that support the findings of this study are available in the supplementary material of this article.
