# Supplementary material for: RSPO2 Coordinates with GDF9:BMP15 Heterodimers to Promote Granulosa Cell and Oocyte Development in Mice
Source: Adv Sci (Weinh). 2025 Jun 10;12(30):e01973. doi: 10.1002/advs.202501973 (PMC12376590; doi:10.1002/advs.202501973)
Supplement: Supplementary file 1 — Supporting Information [file ADVS-12-e01973-s006.docx]

Supporting Information

RSPO2 Coordinates with GDF9:BMP15 Heterodimers to Promote Granulosa Cell and Oocyte Development in Mice

*Yingmei Wang*, *Hongjiang Li*, *Liji You*, *Shuhui Wang*, *Jinglei Bie*, Ziyang Su, *Lanying Shi*, and *You-Qiang Su**


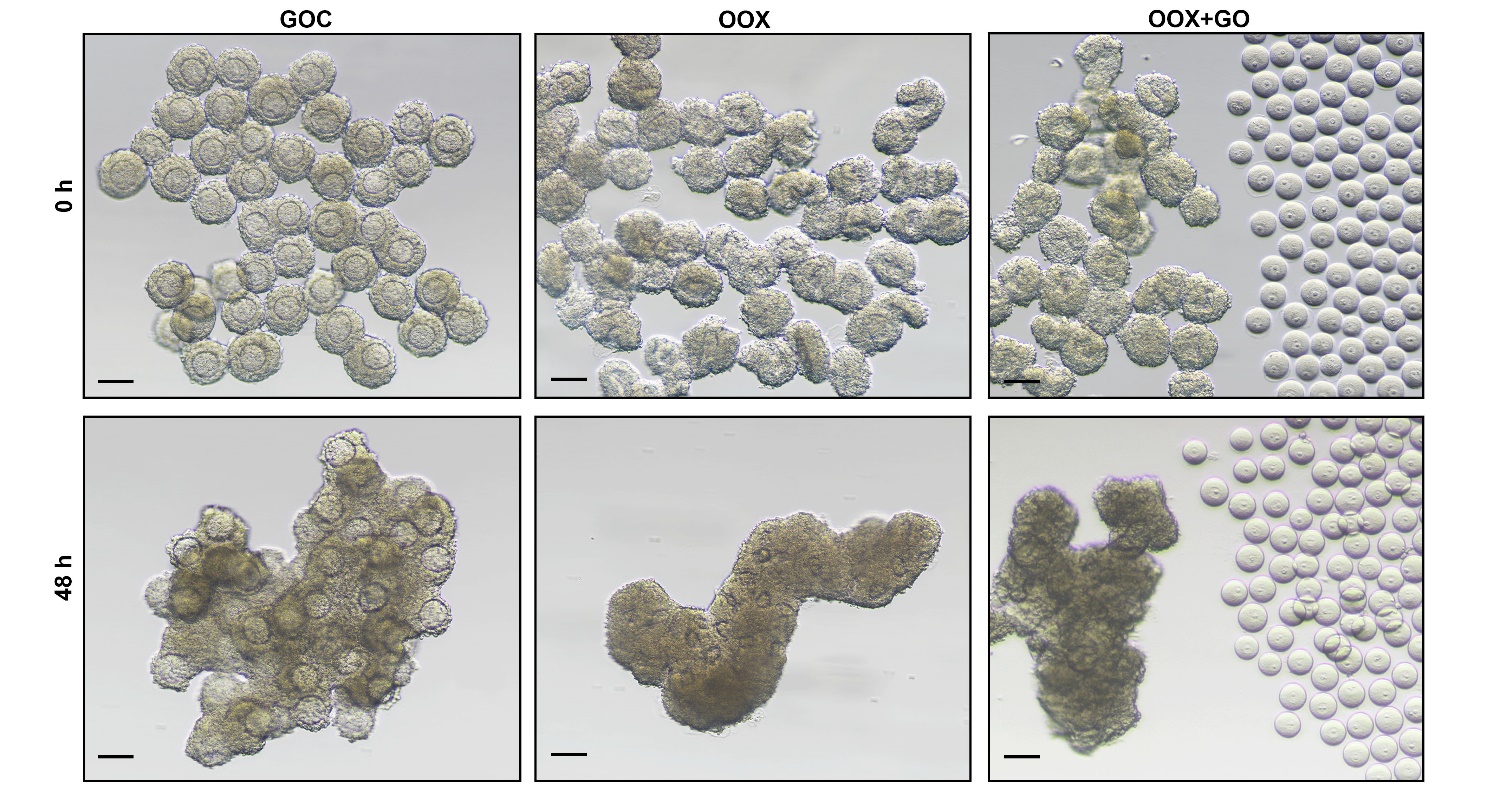


**Figure S1.** Representative micrographs of granulosa cell-oocyte complexes (GOCs), oocytectomized GOCs (OOX), and OOX co-cultured with growing oocytes (OOX+GO) before and after 48 hours of culture. Scale bars = 100 μm.


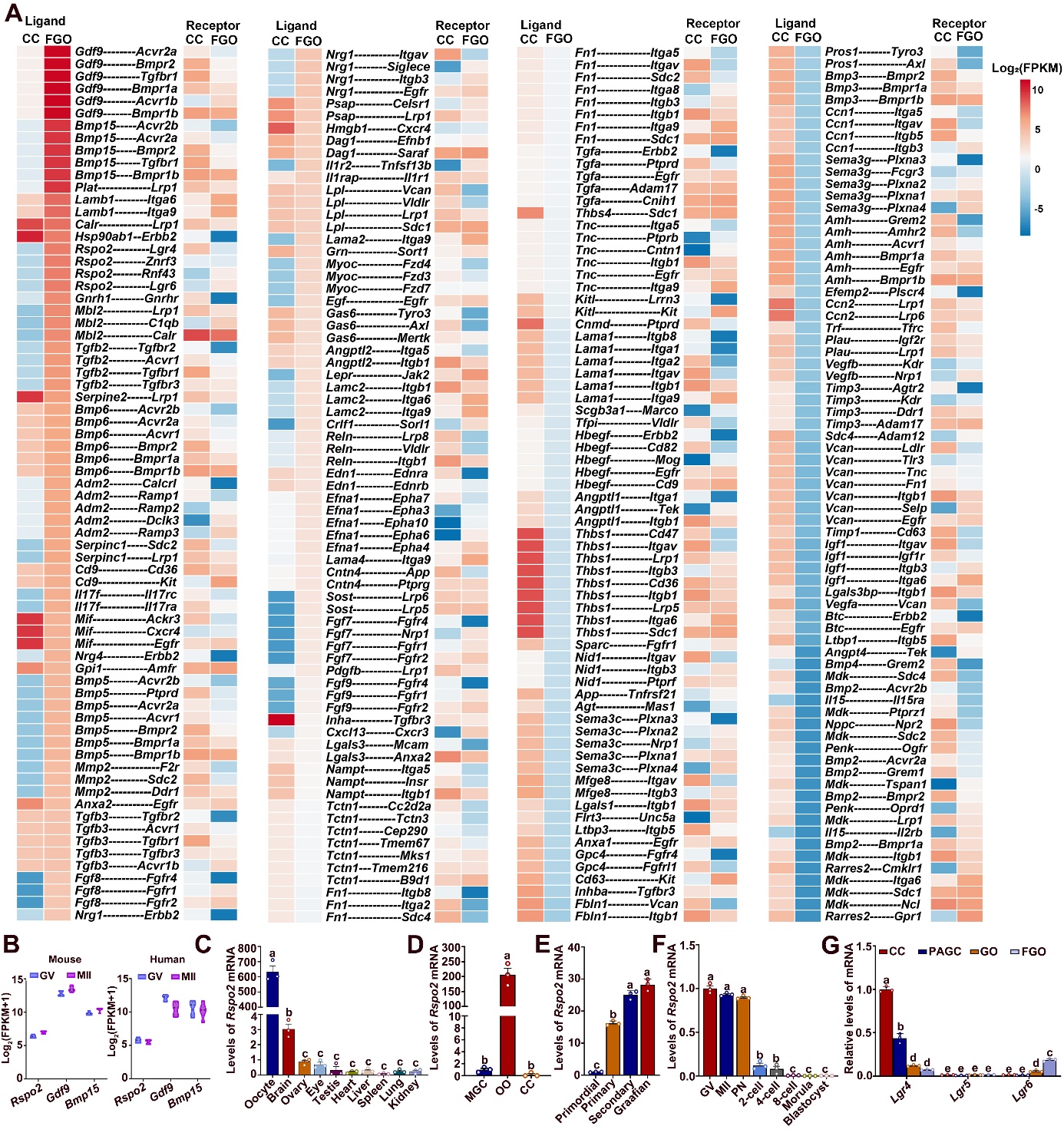
 **Figure S2.** **Potential receptor-ligand pairs expressed by oocytes and cumulus cells. (A)** Heatmap illustrating the expression levels of genes encoding ligands and their cognate receptors in mouse fully-grown oocytes (FGOs) and cumulus cells (CCs) as detected by RNA-Seq. **(B)** Expression levels of *Rspo2*, *Gdf9*, and *Bmp15* in mouse and human GV- and MII-stage oocytes, extracted from publicly available RNA-seq datasets. **(C-F)** Quantitative RT-PCR analysis of *Rspo2* mRNA expression: (C) in various mouse tissues and oocytes; (D) in mural granulosa cells (MGCs), cumulus cells (CCs), and fully-grown oocytes (FGOs) from large antral follicles; (E) in oocytes from primordial, primary, secondary, and Graafian follicles; and (F) in GV- and MII-stage oocytes and preimplantation embryos at different developmental stages. Data are presented as Mean ± SEM (*N*=3). Bars marked with different letters are significantly different, **P*<0.05. **(G)** Quantitative RT-PCR analysis of *Lgr4*, *Lgr5*, and *Lgr6* expression in cumulus cells (CCs), preantral follicle granulosa cells (PAGCs), growing oocytes (GOs), and fully-growing oocytes (FGOs). Data are presented as Mean ± SEM (*N*=3). Groups marked with different letters are significantly different, **P*<0.05.

**
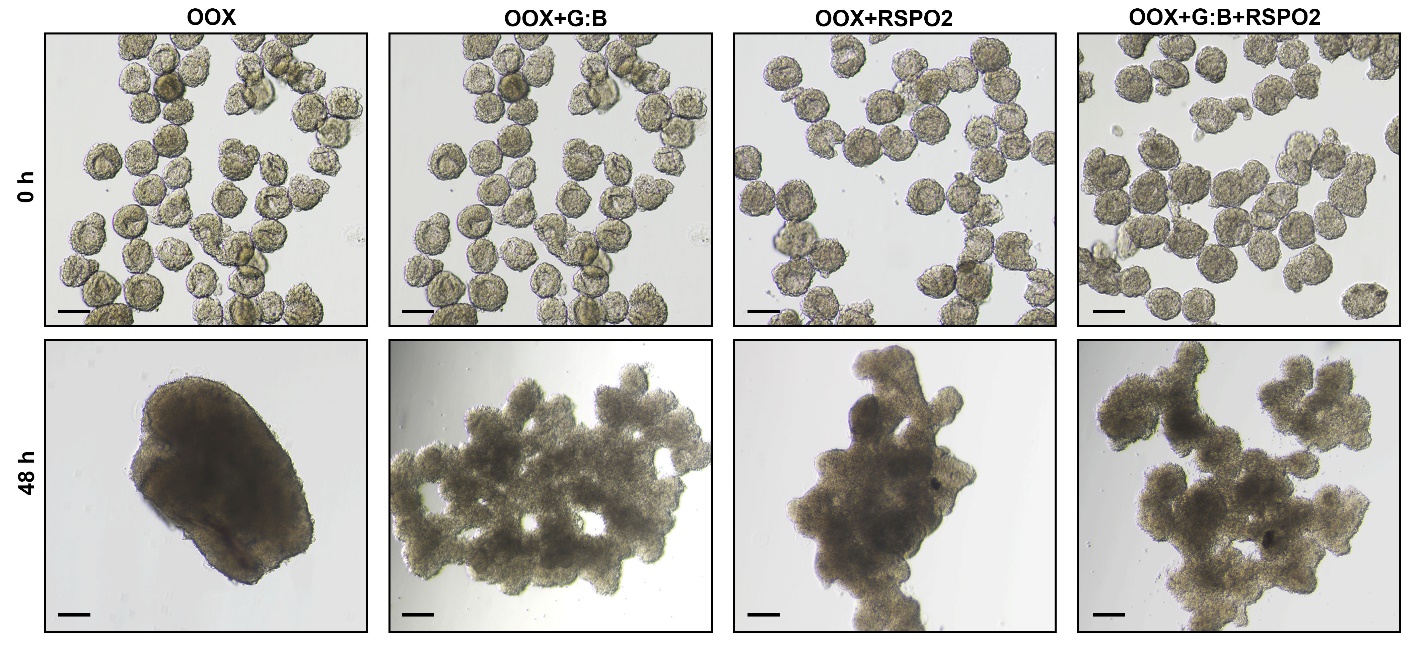
**

**Figure S3.** Representative microphotographs of ocytectomized granulosa cell-oocyte complexes GOCs (OOX), OOX treated with GDF9:BMP15 heterodimers (OOX+G:B), RSPO2 (OOX+RSPO2), and their combination (OOX+G:B+R) before and after 48 hours of culture. Scale bars = 100 μm.


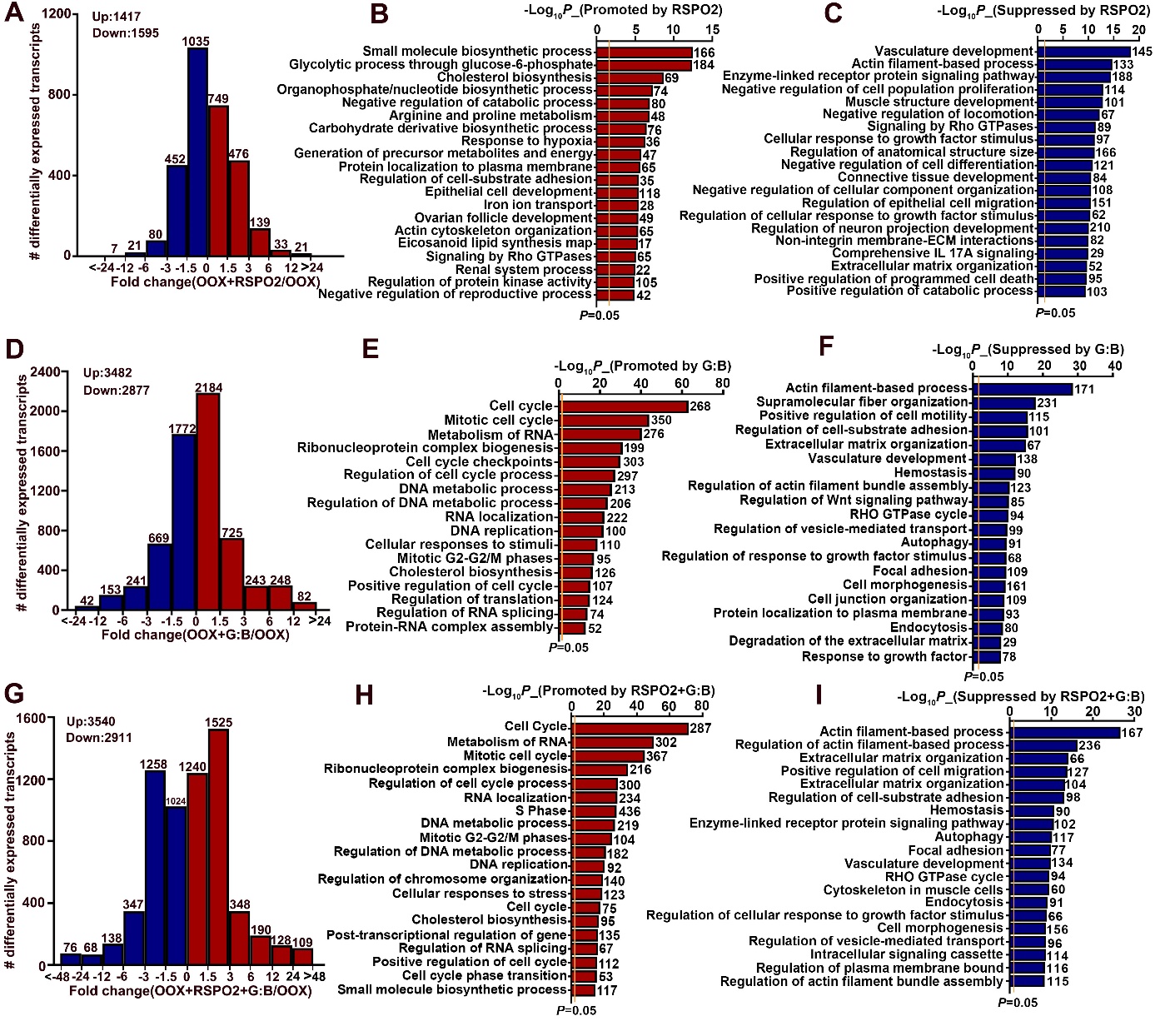


**Figure S4. Gene enrichment analysis of differentially expressed genes (DEGs) in preantral follicle granulosa cells following treatments with RSPO2, GDF9:BMP15 heterodimers (G:B), and their combination (RSPO2+G:B), as detected by RNA-Seq. (A)** The bar graph shows the distribution of differentially expressed genes (DEGs) at various magnitudes of expression level differences between oocytectomized granulosa cell-oocyte complexes (OOXs) treated with and without RSPO2 (OOX+RSPO2 vs OOX). The number of genes in each category is indicated above each bar. **(B-C)** Bar graphs showing the enriched pathways and biological processes associated with genes upregulated (C) and downregulated (D) by RSPO2. **(D)** The bar graph illustrates the distribution of DEGs at various expression level magnitudes between OOXs treated with or without GDF9:BMP15 heterodimers (OOX+G:B vs OOX). The number of genes in each category is indicated above the corresponding bars. **(E-F)**The bar graphs show the enriched pathways and biological processes linked to genes promoted (E) and suppressed (F) by G:B, respectively. **(G)** The bar graph represents the distribution of DEGs at various magnitudes of expression level differences between OOXs treated with and without RSPO2+G:B (OOX+RSPO2+G:B vs OOX). The number of genes in each category is indicated above each bar. **(H-I)** The bar graphs highlight the enriched pathways and biological processes associated with genes promoted (H) and suppressed (I) by RSPO2+G:B, respectively.


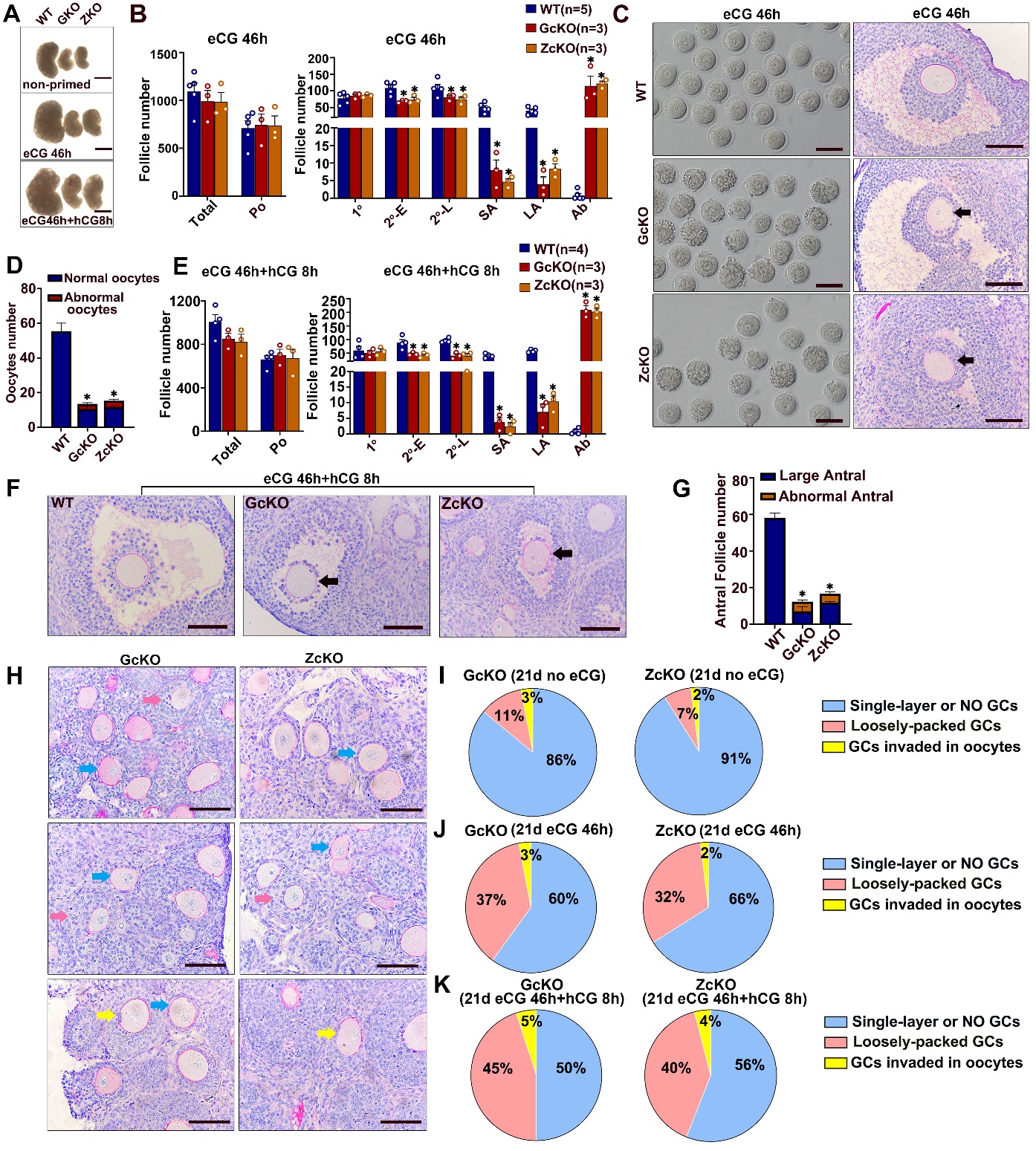


**Figure S5. Defective follicular development in *Rspo2*-GcKO and *Rspo2*-ZcKO ovaries. (A)** Representative micrographs comparing ovarian size between wild-type (WT) and *Rspo2*-GcKO (GcKO), and *Rspo2*-ZcKO (ZcKO) mice. Scale bars = 100 µm. (**B**) Quantification of the number of follicles at different developmental stages in 21-day-old WT, *Rspo2*-GcKO, and *Rspo2*-ZcKO females following priming with eCG (46 hours). Follicle stages are cclassified as: Po (primordial follicles), 1^o^ (primary follicles), 2^o^-E (early secondary follicles), 2^o^-L (late secondary follicles), SA, (small antral follicles), LA (large antral follicles), and Ab (abnormal follicles). Data are presented as Mean ± SEM (WT, *N*=5; *Rspo2*-GcKO and *Rspo2*-ZcKO, *N*=3). **P*<0.05, cKO vs WT. (**C**) Representative micrographs showing the morphology of abnormal oocytes retrieved from antral follicles (left panel) or within the antral follicles (right panel) of eCG-primed *Rspo2*-GcKO (GcKO) and *Rspo2*-ZcKO (ZcKO) female mice. Arrows indicate oocytes with cumulus cells infiltrating the cytoplasm. Scale bars = 100 µm. **(D)** Quantification of abnormal oocytes retrieved from antral follicles of following eCG (46 hours) priming. Data are presented as Mean ± SEM (*N*=3). **P*<0.05, cKO vs WT. **(E)** Quantification of the number of follicles at different stages of development in the 21-day-old WT, *Rspo2*-GcKO and *Rspo2*-ZcKO females after eCG (46 hours) and hCG (8 hours) priming. Follicle stages are classified as: Po (primordial follicles), 1^o^ (primary follicles), 2^o^-E (early secondary follicles), 2^o^-L (late secondary follicles), SA, (small antral follicles), LA (large antral follicles), and Ab (abnormal follicles). Data are presented as Mean ± SEM (WT, *N*=4; *Rspo2*-GcKO and *Rspo2*-ZcKO, *N*=3). **P*<0.05, cKO vs WT. **(F)** Representative micrographs showing abnormal antral follicles with fewer cumulus cells sparsely surrounding the oocyte in *Rspo2*-GcKO (GcKO) and *Rspo2*-ZcKO (ZcKO) ovaries after eCG (46 hours) and hCG (8 hours) priming. Arrows indicate oocytes with sparsely distributed cumulus cells. Scale bars = 100 µm. **(G)** Quantification of abnormal antral follicles in *Rspo2*-GcKO (GcKO) and *Rspo2*-ZcKO (ZcKO) ovaries after eCG (46 hours) and hCG (8 hours) priming. Data are presented as Mean ± SEM (*N*=3) **P*<0.05, cKO vs WT. **(H)** Representative micrographs showing abnormal follicles in ovarian sections of 21-day-old *Rspo2*-GcKO (GcKO) and *Rspo2*-ZcKO (ZcKO) mice that were not primed with eCG or hCG. Blue arrows indicate follicles with single-layer or no granulosa cells, red arrows indicate follicles with loosely-packed granulosa cells, and yellow arrows indicate follicles with granulosa cells infiltrating the cytoplasm. Scale bars = 100 µm. (**I-K**) Quantification of the proposition of different types of abnormal follicles in the ovaries of 21-day-old *Rspo2*-GcKO (GcKO) and *Rspo2*-ZcKO (ZcKO) females following priming with or without eCG (46 hours) and hCG (8 hours).


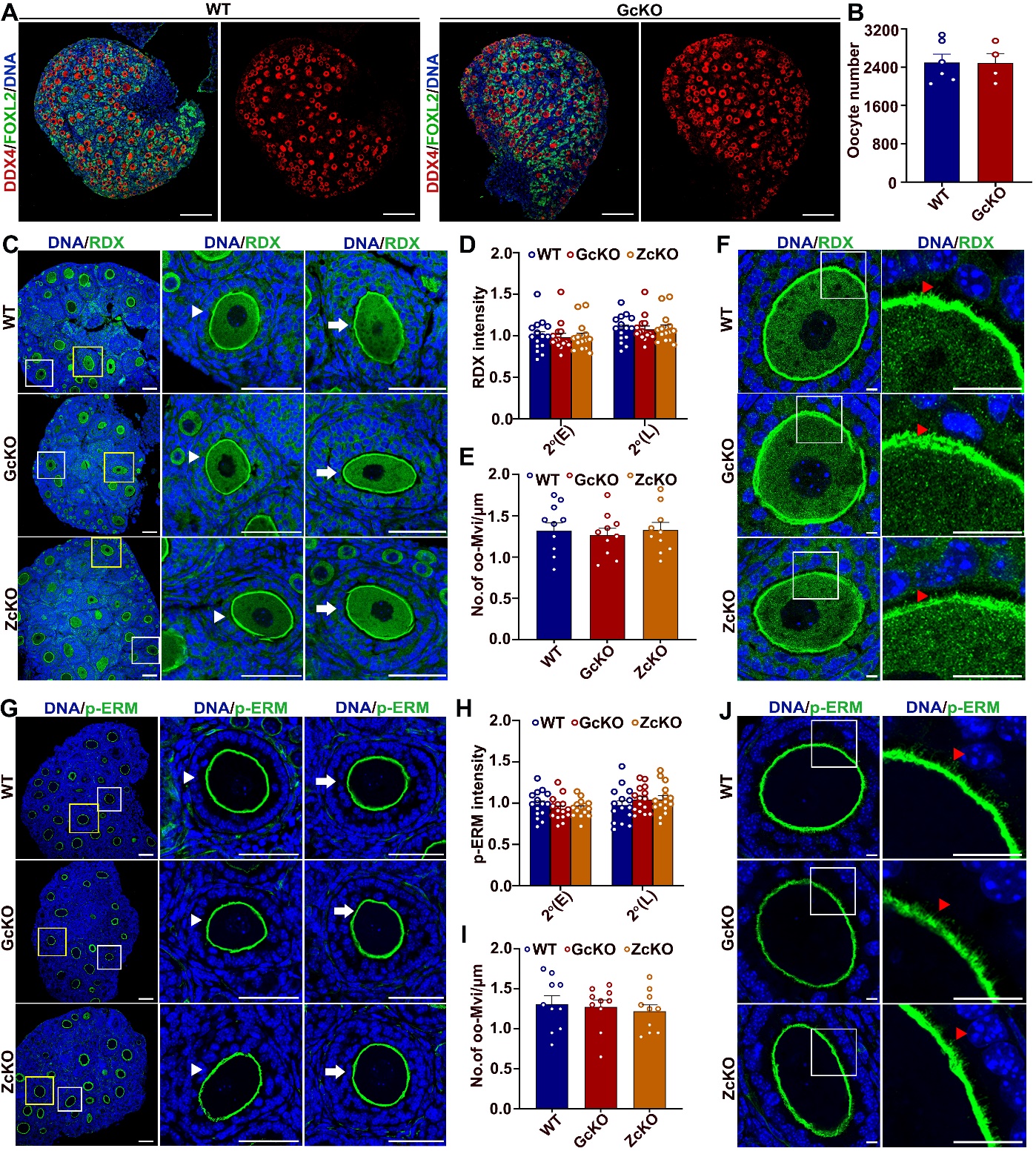


**Figure S6. Unaltered primordial follicle pool establishment and oocyte microvilli number in *Rspo2*-cKO Mice** (**A**) Immunofluorescece (IF) staining for DDX4 (red) and FOXL2 (green) on ovarian sections from 3-day-old wild-type (WT) and *Rspo2*-GcKO (GcKO) females. DNA is counterstained with DAPI (blue). Scale bars = 100 µm. (**B**) Quantitative analysis of DDX4-positive oocytes in 3-day-old wild-type (WT, *N*=6) and *Rspo2*-GcKO (GcKO, *N*=4) females. Data are presented as Mean ± SEM. There is no significant difference between *Rspo2*-GcKO and WT. **(C)** Immunofluorescent (IF) staining for RDX (green) in ovarian sections from 12-day-old WT, *Rspo2*-GcKO (GcKO), and *Rspo2*-ZcKO (ZcKO) females. Enlarged views of the boxed areas are shown on the right. Arrowheads indicate early secondary follicles, and arrows indicate late secondary follicles. DNA is counterstained with DAPI (blue). Scale bars = 100 µm. **(D)** Quantification of RDX fluorescence intensity in early secondary (2^o^(E)) and late secondary(2^o^(L)) follicles. A total of 15 follicles per stage were analyzed for each genotype. Data are presented as Mean ± SEM. No significant difference was observed between *Rspo2*-cKO and WT. **(E)** Quantification of the number of oocyte microvilli (Oo-Mvi) per micrometer in oocytes from late secondary follicles (2–3 granulosa cell layers), based on IF staining on RDX. Ten follicles per genotype were analyzed. Data are presented as Mean ± SEM. No significant difference was observed between *Rspo2*-cKO and WT. (**F**) Representative IF micrographs showing RDX staining. The magnified view of the boxed area (right panel) highlights oocyte microvilli (indicated by red arrowheads). Scale bars = 10 µm. (**G**) IF staining for p-ERM (green) on ovarian sections from 12-day-old WT, *Rspo2*-GcKO (GcKO), and *Rspo2*-ZcKO (ZcKO) females. Enlarged views of the boxed area are shown on the right, with arrowheads and arrows indicating early secondary and late secondary follicles, respectively. DNA is counterstained with DAPI (blue). Scale bars = 100 µm. **(H)** Quantification of p-ERM intensity in secondary (2^o^(E)) and late secondary(2^o^(L)) follicles. from 12-day-old wild-type (WT), *Rspo2*-GcKO (GcKO), and *Rspo2*-ZcKO (ZcKO) females. A total of 15 early secondary and 15 late secondary follicles were counted for each genotype. Data are presented as Mean ± SEM. No significant difference was observed between *Rspo2*-cKO and WT. **(I)** Quantification of the number of oocyte microvilli (Oo-Mvi) per micrometer in oocytes from late secondary follicles (2–3 granulosa cell layers), based on the staining on p-ERM. Ten follicles per genotype were analyzed. Data are presented as Mean ± SEM. No significant difference was observed between *Rspo2*-cKO and WT. (**J**) Representative IF micrographs of p-ERM staining. The magnified view of the boxed areas (right panel) highlight oocyte microvilli (indicated by red arrowheads), Scale bars indicate 10 µm.


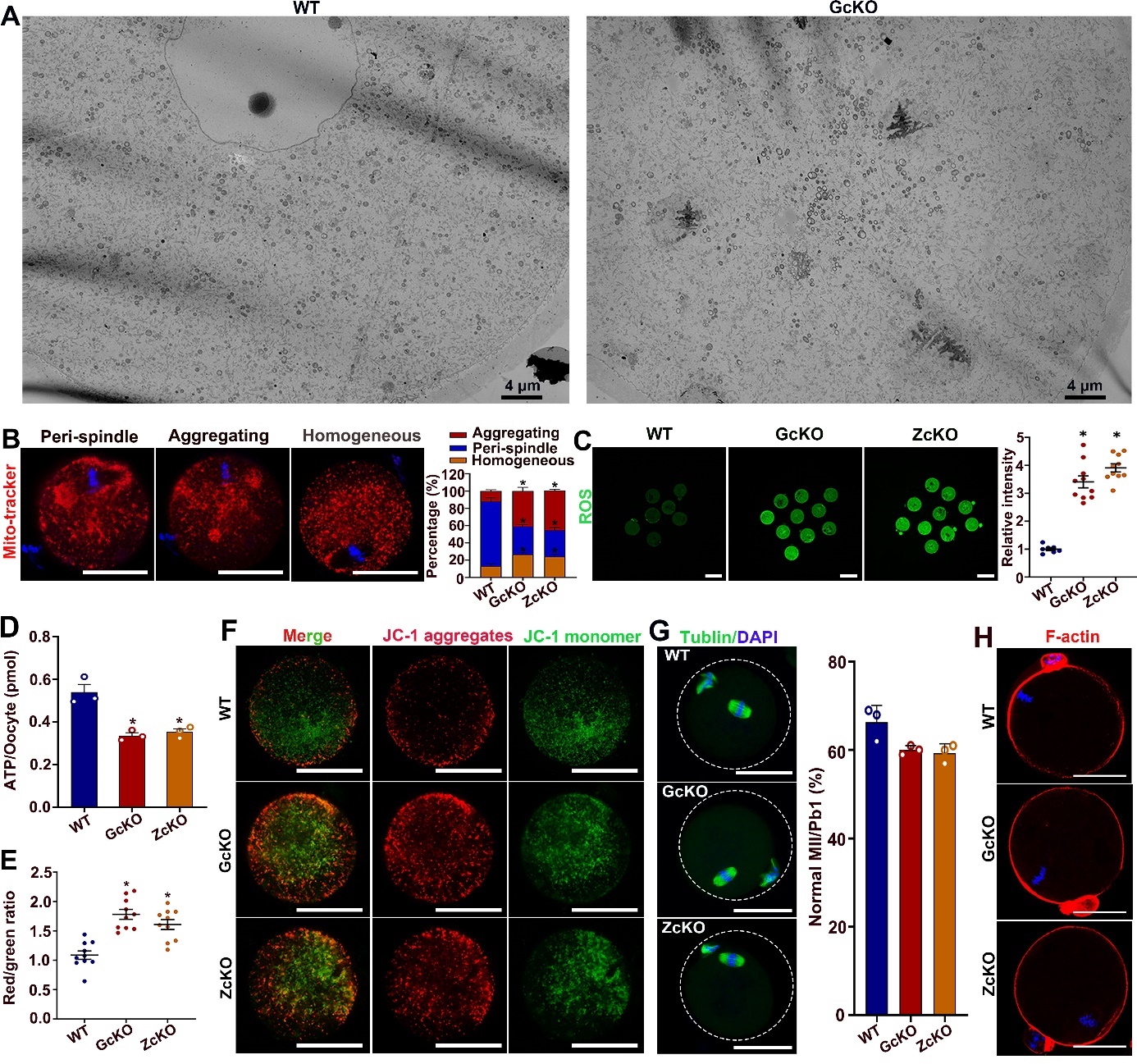


**Figure S7.** **Impaired mitochondrial distribution and function in the MII-stage oocytes of *Rspo2* oocyte-specific knockout mice.** (**A**) Representative transmission electron microscopy (TEM) micrographs showing mitochondrial distribution in wild-type (WT) and *Rspo2*-GcKO MII-stage oocytes. (**B**) Analysis of changes in mitochondrial distribution in *Rspo2*-cKO MII-stage ooocytes by Mitotracker labeling (red) and live imaging. Chromosomes are labeled with Hoechst (blue). The left panel displays representative micrographs showing three patterns of mitochondrial distribution: “peri-spindle” (mitochondria forming aggregates surrounding the spindle), “aggregating” (mitochondria forming clusters in the cytoplasm), and “homogeneous” (mitochondria distributed evenly throughout the cytoplasm). Scale bar=50 μm. The right bar graph quantifies the ratio of oocytes exhibiting different patterns of mitochondrial distribution. The experiment was repeated independently three times, with 10 oocytes per genotype in each experiment. Data are presented as Mean ± SEM. **P*<0.05, *Rspo2*-cKO vs WT by χ² analysis. **(C)** Evaluation of reactive oxygen species (ROS) levels in *Rspo2*-cKO and WT MII-stage oocytes. The left panel show representative micrographs of ROS staining (green) in oocytes. Scale bars=100 µm. The right bar graph quantifies ROS levels in the *Rspo2*-cKO oocytes. A total of 10 oocytes were evaluated for each genotype. Data are presented as Mean ± SEM. **P*<0.05, *Rspo2*-cKO vs WT. **(D)** Comparison of ATP levels between *Rspo2*-cKO and WT MII-stage oocytes. The experiment was repeated independently three times, with 50 oocytes per genotype in each experiment. Data are presented as Mean ± SEM. **P*<0.05, *Rspo2*-cKO vs WT. **(E, F)** Evaluation of mitochondrial membrane potential levels in the *Rspo2*-cKO and WT MII-stage ooocytes by JC1 staining. (E) The bar graph quantifies mitochondrial membrane potential levels in *Rspo2*-cKO oocytes. A total of 10 oocytes were evaluated. Data are presented as Mean ± SEM. **P*<0.05, *Rspo2*-cKO vs WT. **(F)** Representative micrographs show oocytes labeled with the JC1 probe. Scale bars=100 µm. (**G**) Confocal microscopic analysis of spindle morphology and chromosome alignment in matured oocytes. The left panel presents representative micrographs of IF staining for tubulin (green) and chromosome (blue). Scale bars = 50 µm. The right panel quantifies the percentage of oocytes reaching normal MII stage. Experiments were repeated three times independently, with 15 oocytes for each genotype were evaluated. Data are presented as Mean ± SEM. **P*<0.05, *Rspo2*-cKO vs WT. (**H**) F-actin staining (red) showing the cytoskeleton of MII-stage oocytes from WT, *Rspo2*-GcKO and *Rspo2*-ZcKO mice. Chromosomes are counterstained with DAPI (blue). Scale bars = 50 µm. **P* < 0.05, compared with WT.

**
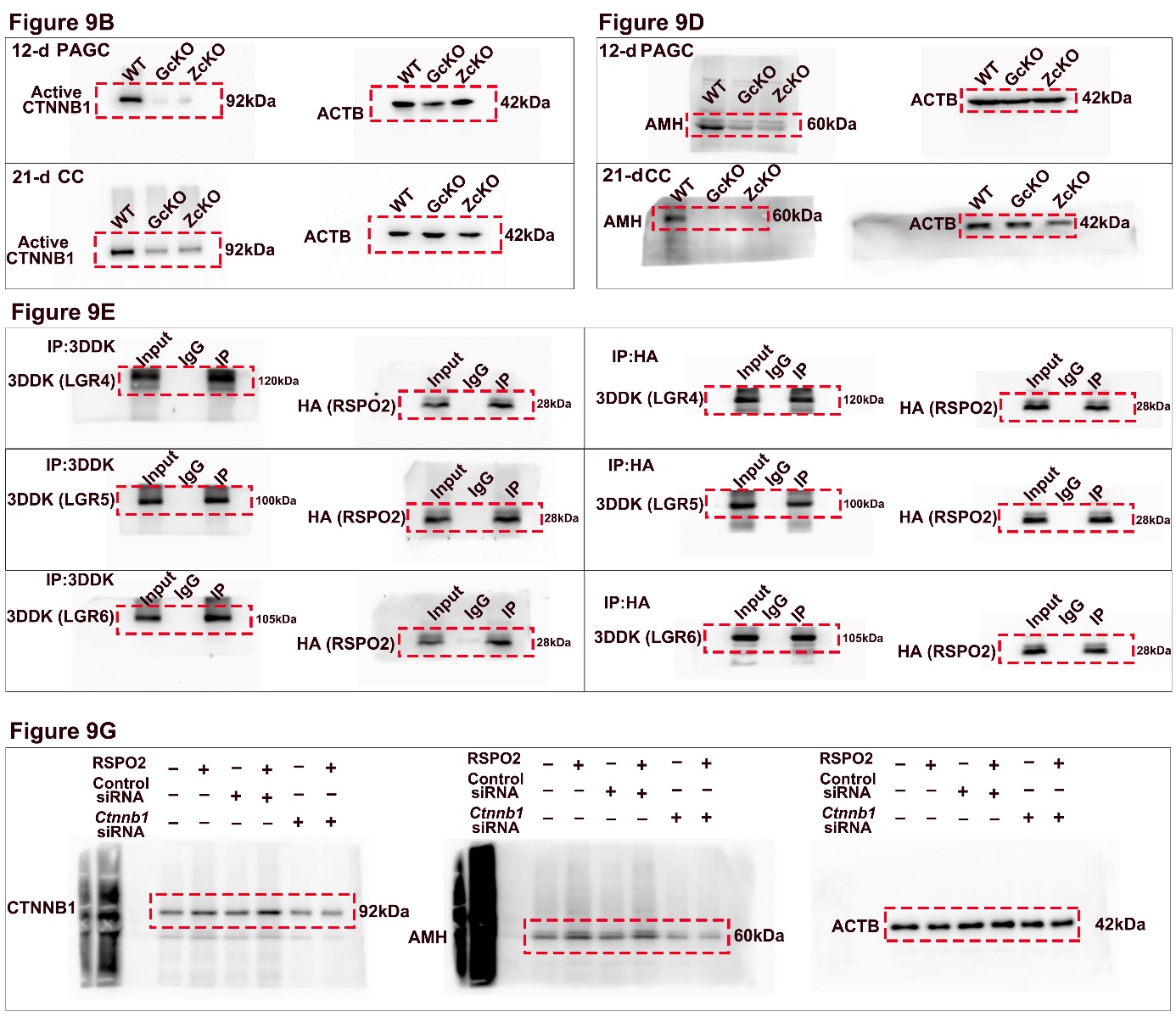
**

**Figure S8.** **The original, unaltered blot images corresponding to Figure 9.**


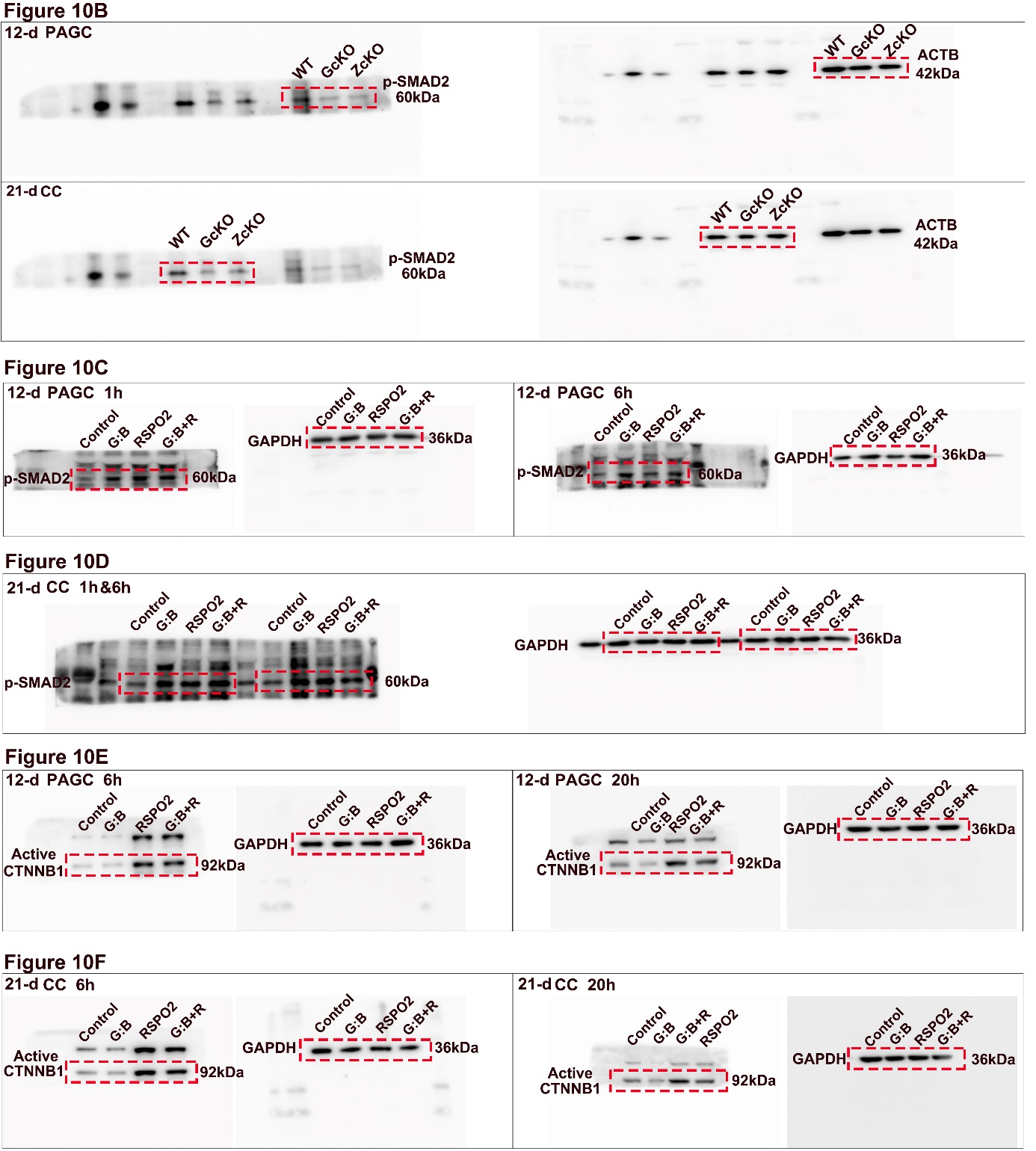


**Figure S9.** **The original, unaltered blot images corresponding to Figure 10.**

**Table S1. Primers used for PCR analyses**

| **Gene** | **Forward primer sequence(5'-3')** | **Reverse primer sequence(5'-3')** | **Type of application** |
| --- | --- | --- | --- |
| ***Acat2*** | CCCGTGGTCATCGTCTCAG | GGACAGGGCACCATTGAAGG | qRT-PCR |
| ***Acta2*** | GTCCCAGACATCAGGGAGTAA | TCGGATACTTCAGCGTCAGGA | qRT-PCR |
| ***Acvr1*** | GTGGAAGATTACAAGCCACCA | GGGTCTGAGAACCATCTGTTAGG | qRT-PCR |
| ***Ahr*** | AGCCGGTGCAGAAAACAGTAA | AGGCGGTCTAACTCTGTGTTC | qRT-PCR |
| ***Akt3*** | TGGGTTCAGAAGAGGGGAGAA | AGGGGATAAGGTAAGTCCACATC | qRT-PCR |
| ***Amh*** | CCCAGGTCACAGTCACAGG | TCCACGGTTAGCACCAAAT | qRT-PCR |
| ***Amhr2*** | GGGGCTTTGGACACTGCTT | GTCTCGGCATCCTTGCATCTC | qRT-PCR |
| ***Angpt2*** | GGTTGCTATCCGTAAAGAAGAGC | GGGGAAGGTCAGTGTGTAGATG | qRT-PCR |
| ***Ar*** | GCTTCTGGCTGTCACTACGG | CAAGATGGGCAATTTTTCCT | qRT-PCR |
| ***Asns*** | GCAGTGTCTGAGTGCGATGAA | TCTTATCGGCTGCATTCCAAAC | qRT-PCR |
| ***Atp5k*** | GTTCAGGTCTCTCCACTCATCA | CGGGGTTTTAGGTAACTGTAGC | qRT-PCR |
| ***Bmp2*** | TCTGTACCGCAGGCACTCA | CCGTTTTCCCACTCATCTC | qRT-PCR |
| ***Bmpr1b*** | CCCTCGGCCCAAGATCCTA | CAACAGGCATTCCAGAGTCATC | qRT-PCR |
| ***Cav2*** | TTGGGATAGGTGAGAGTCGAAT | TGTTTCACAAGATTGATGTCCCC | qRT-PCR |
| ***Ccn2*** | GGGCCTCTTCTGCGATTTC | ATCCAGGCAAGTGCATTGGTA | qRT-PCR |
| ***Ccnd2*** | GAGTGGGAACTGGTAGTGTTG | CGCACAGAGCGATGAAGGT | qRT-PCR |
| ***Cdh11*** | CTGGGTCTGGAACCAATTCTTT | GCCTGAGCCATCAGTGTGTA | qRT-PCR |
| ***Cdkn1a*** | CCTGGTGATGTCCGACCTG | CCATGAGCGCATCGCAATC | qRT-PCR |
| ***Cdkn1c*** | CGAGGAGCAGGACGAGAATC | GAAGAAGTCGTTCGCATTGGC | qRT-PCR |
| ***Cited2*** | CGCCAGGTTTAACAACTCCCA | TGCTGGTTTGTCCCGTTCAT | qRT-PCR |
| ***Cldn11*** | ATGGTAGCCACTTGCCTTCAG | AGTTCGTCCATTTTTCGGCAG | qRT-PCR |
| ***Cldn5*** | GCAAGGTGTATGAATCTGTGCT | GTCAAGGTAACAAAGAGTGCCA | qRT-PCR |
| ***Col3a1*** | CTGTAACATGGAAACTGGGGAAA | CCATAGCTGAACTGAAAACCACC | qRT-PCR |
| ***Cox4i2*** | CTGCCCGGAGTCTGGTAATG | CAGTCAACGTAGGGGGTCATC | qRT-PCR |
| ***Cox6b2*** | ATGAGCAGATCAAGCAGGGAAC | CACAAAGACAAAGTGGAGGGAG | qRT-PCR |
| ***Cox7a2*** | GCTGGCCCTTCGTCAGATT | GGCATCCCATTATCCTCCTGAA | qRT-PCR |
| ***Cox7c*** | GCCATTTCTTCCGCCTTCC | CCGCCACTTGTTTTCCACT | qRT-PCR |
| ***Cxcl12*** | TGCATCAGTGACGGTAAACCA | TTCTTCAGCCGTGCAACAATC | qRT-PCR |
| ***Cxcr4*** | GACTGGCATAGTCGGCAATG | AGAAGGGGAGTGTGATGACAAA | qRT-PCR |
| ***Cyp19a1*** | CGGAGGAATGCACAGGCTCGAG | CGATGTACTTCCCAGCACAGC | qRT-PCR |
| ***Dhcr7*** | AGGCTGGATCTCAAGGACAAT | GCCAGACTAGCATGGCCTG | qRT-PCR |
| ***Dhh*** | AACCACATCCACGTATCGGT | GGTCCAGGAAGAGCAGCACT | qRT-PCR |
| ***Dkkl1*** | GACCTGCTACGAGACCTGGA | CTGGAGAGGGTATGGTTGCC | qRT-PCR |
| ***Egr1*** | TCGGCTCCTTTCCTCACTCA | CTCATAGGGTTGTTCGCTCGG | qRT-PCR |
| ***Eln*** | TTGCTGATCCTCTTGCTCAAC | GCCCCTGGATAATAGACTCCAC | qRT-PCR |
| ***Esr1*** | CTGCCAAGGAGACTCGCTACTG | ATGCCCACTTCGTAACACTTGC | qRT-PCR |
| ***Fdps*** | GGAGGTCCTAGAGTACAATGCC | AAGCCTGGAGCAGTTCTACAC | qRT-PCR |
| ***Fdx1*** | TAACAGTCCACTTCAAGAACCGA | CACAACATCTAGCAGAGAGTCG | qRT-PCR |
| ***Fn1*** | ATGTGGACCCCTCCTGATAGT | GCCCAGTGATTTCAGCAAAGG | qRT-PCR |
| ***Fos*** | CGGGTTTCAACGCCGACTA | TTGGCACTAGAGACGGACAGA | qRT-PCR |
| ***Foxl2*** | CAACACCGGAGAAACCAGACC | GCGGCACCTTGATGAAGCACT | qRT-PCR |
| ***Fshr*** | ATCATTTTCTGGATTTGGGGAC | AATTTCATGCAAGTTGGGTAGG | qRT-PCR |
| ***Fst*** | TGCTGCTACTCTGCCAGTTC | GTGCTGCAACACTCTTCCTTG | qRT-PCR |
| ***Fzd8-F*** | ATGGAGTGGGGTTACCTGTTG | CACCGTGATCTCTTGGCAC | qRT-PCR |
| ***Gadd45b*** | CAACGCGGTTCAGAAGATGC | GGTCCACATTCATCAGTTTGGC | qRT-PCR |
| ***Gja1*** | ACAGCGGTTGAGTCAGCTTG | GAGAGATGGGGAAGGACTTGT | qRT-PCR |
| ***Grb14*** | TGCTGCTCTCCGCTTACATC | AGGTGCTCAAAAAGGGTCCAG | qRT-PCR |
| ***Grem2*** | GGTAGCTGAAACACGGAAGAA | TCTTGCACCAGTCACTCTTGA | qRT-PCR |
| ***Hif1a*** | ACCTTCATCGGAAACTCCAAAG | ACTGTTAGGCTCAGGTGAACT | qRT-PCR |
| ***Hmgcs1*** | AACTGGTGCAGAAATCTCTAGC | GGTTGAATAGCTCAGAACTAGCC | qRT-PCR |
| ***Hsd11b2*** | GGTTGTGACACTGGTTTTGGC | AGAACACGGCTGATGTCCTCT | qRT-PCR |
| ***Hspg2*** | TTCCAGATGGTCTATTTCCGGG | CTTGGCACTTGCATCCTCC | qRT-PCR |
| ***Hspg2*** | TTCCAGATGGTCTATTTCCGGG | CTTGGCACTTGCATCCTCC | qRT-PCR |
| ***Id2*** | ATGAAAGCCTTCAGTCCGGTG | AGCAGACTCATCGGGTCGT | qRT-PCR |
| ***Ifitm3*** | CCCCCAAACTACGAAAGAATCA | ACCATCTTCCGATCCCTAGAC | qRT-PCR |
| ***Igf1*** | GTGAGCCAAAGACACACCCA | ACCTCTGATTTTCCGAGTTGC | qRT-PCR |
| ***Ihh*** | CTCTTGCCTACAAGCAGTTCA | CCGTGTTCTCCTCGTCCTT | qRT-PCR |
| ***Il17d*** | AGCACACCCGTCTTCTCTC | GCTGGAGTTCGCACTGTCC | qRT-PCR |
| ***Inha*** | AAGGTGGGGATCCTGGAAT | CTGGTGGCTGCGTATGTGT | qRT-PCR |
| ***Inhbb*** | TCCGAGATCATCAGCTTTG | TAGGTTCTGGTTGCCTTCA | qRT-PCR |
| ***Irf1*** | ATGCCAATCACTCGAATGCG | TTGTATCGGCCTGTGTGAATG | qRT-PCR |
| ***Jup*** | TGGCAACAGACATACACCTACG | GGTGGTAGTCTTCTTGAGTGTG | qRT-PCR |
| ***Kitl*** | AGACATTACAAAACTGGTG | GAAGAGTAGTCAAGCTGAG | qRT-PCR |
| ***Lgr4*** | TACAACTGGCTGGTAACGACC | TTGAGTTCTTTCAACCCAGACAA | qRT-PCR |
| ***Lgr5*** | CCTACTCGAAGACTTACCCAGT | GCATTGGGGTGAATGATAGCA | qRT-PCR |
| ***Lgr6*** | GAGGACGGCATCATGCTGTC | GCTCCGTGAGGTTGTTCATACT | qRT-PCR |
| ***Myc*** | CCCTATTTCATCTGCGACGAG | GAGAAGGACGTAGCGACCG | qRT-PCR |
| ***Ndufa3*** | ATGGCCGGGAGAATCTCTG | AGGGGCTAATCATGGGCATAAT | qRT-PCR |
| ***Ndufc1*** | GTAGTGCTGCGCTCGTTTTC | CCAACCAGTTAGGTTTGGCAT | qRT-PCR |
| ***Ndufs6*** | ACTTTGCCATTGATTTGATAGC | TCCCCGTTTTTGTTTCTTTGTC | qRT-PCR |
| ***Nfkb1*** | ATGGCAGACGATGATCCCTAC | TGTTGACAGTGGTATTTCTGGTG | qRT-PCR |
| ***Nos2*** | GTTCTCAGCCCAACAATACAAGA | GTGGACGGGTCGATGTCAC | qRT-PCR |
| ***Nppc*** | CAGAAAAAGGGTGACAAGACTCC | ATCCCAGACCGCTCATGGA | qRT-PCR |
| ***Npr1*** | GCTTGTGCTCTATGCAGATCG | TCGACGAACTCCTGGTGATTTA | qRT-PCR |
| ***Nr4a1*** | TTGAGTTCGGCAAGCCTACC | GTGTACCCGTCCATGAAGGTG | qRT-PCR |
| ***Nrp2*** | GCTGGCTACATCACTTCCCC | CAATCCACTCACAGTTCTGGTG | qRT-PCR |
| ***Pdgfrb*** | TTCCAGGAGTGATACCAGCTT | AGGGGGCGTGATGACTAGG | qRT-PCR |
| ***Pik3cd*** | GTAAACGACTTCCGCACTAAGA | GCTGACACGCAATAAGCCG | qRT-PCR |
| ***Pik3ip1*** | ATGCAGCGAATCACCTTGC | TTTCATCCACGGTCTCACAGG | qRT-PCR |
| ***Plk1*** | CCCGCTGGCGAAAGAAATTC | CATTTGGCGAAGCCTCCTTTA | qRT-PCR |
| ***Psat1*** | AAGCCACCAAGCAAGTGGTTA | GATGCCGAGTCCTCTGTAGTC | qRT-PCR |
| ***Rorc*** | GACCCACACCTCACAAATTGA | AGTAGGCCACATTACACTGCT | qRT-PCR |
| ***Rspo2*** | GTTGCAGCCGATGTCAACAG | CGGTGTCCATAATACCCTGATG | qRT-PCR |
| ***Slc1a4*** | GGCATCGCTGTTGCTTACTTC | CGAGGAAAGAGTCCACTGTCT | qRT-PCR |
| ***Slc38a3*** | CGAGGGGAAGACATCATTTG | CGAAGACTTGAGGAGCAGGT | qRT-PCR |
| ***Slc7a11*** | GGCACCGTCATCGGATCAG | CTCCACAGGCAGACCAGAAAA | qRT-PCR |
| ***Smad3*** | AACACTAACTTCCCTGCT | TGGTTTCTCCATCTTCAC | qRT-PCR |
| ***Smad7*** | GGCCGGATCTCAGGCATTC | TTGGGTATCTGGAGTAAGGAGG | qRT-PCR |
| ***Socs3*** | ATGGTCACCCACAGCAAGTTT | TCCAGTAGAATCCGCTCTCCT | qRT-PCR |
| ***Sox4*** | CGGCTGCATCGTTCTCTCC | GGTAGACGTGCTTCACTTTCTTG | qRT-PCR |
| ***Tgfb1*** | CCACCTGCAAGACCATCGAC | CTGGCGAGCCTTAGTTTGGAC | qRT-PCR |
| ***Tgif2*** | ATGTCGGACAGCGATCTAGG | TCCCGGAGGATCTTTACTGAC | qRT-PCR |
| ***Tnfsf12*** | TGTGGTACCTTTCTTGGA | GTTGATTTTGGTCTCTTC | qRT-PCR |
| ***Tpbg-F*** | CGGCAACCACCTGAAGGAA | AAGGCCACCATACCCTCGAA | qRT-PCR |
| ***Trp53inp1*** | AAGTGGTCCCAGAATGGAAGC | GGCGAAAACTCTTGGGTTGT | qRT-PCR |
| ***Uqcrb*** | GGCCGATCTGCTGTTTCAG | CATCTCGCATTAACCCCAGTT | qRT-PCR |
| ***Vegfa*** | GCACATAGAGAGAATGAGCTTCC | CTCCGCTCTGAACAAGGCT | qRT-PCR |
| ***Vim*** | CGTCCACACGCACCTACAG | GGGGGATGAGGAATAGAGGCT | qRT-PCR |
| ***Wnt5b*** | CAGGGCATTGGGATGGGTTG | AGGAAGTTGGCTGCACACGG | qRT-PCR |
| ***Wnt6*** | GCAAGACTGGGGGTTCGAG | CCTGACAACCACACTGTAGGAG | qRT-PCR |
| ***Wt1*** | GAGAGCCAGCCTACCATCC | GGGTCCTCGTGTTTGAAGGAA | qRT-PCR |
| ***Zfp36*** | CCACCTCCTCTCGATACAAGA | GCTTGGCGAAGTTCACCCA | qRT-PCR |
| ***Rspo2-Loxp*(F1R1)** | ACTGAAGGAACAAAAGGGAGAATTG | AGGCACGTTGTTACTGTAGCTT | Genotype |
| ***Gdf9-CRE*** | TCTGATGAAGTCAGGAAGAACC | GAGATGTCCTTCACTCTGATTC | Genotype |
| ***Zp3-CRE*** | GCCTGCATTACCGGTCGATGC | CAGGGTGTTATAAGCAATCCC | Genotype |
| ***Rspo2-*F1R2** | ACTGAAGGAACAAAAGGGAGAATTG | AGACACTTGGACTTCAAGGAAGAT | Genotyping knock out allele |
